# Applicability of Scrape Loading-Dye Transfer Assay for Non-Genotoxic Carcinogen Testing

**DOI:** 10.3390/ijms22168977

**Published:** 2021-08-20

**Authors:** Iva Sovadinová, Brad L. Upham, James E. Trosko, Pavel Babica

**Affiliations:** 1 RECETOX, Faculty of Science, Masaryk University, 625-00 Brno, Czech Republic; iva.sovadinova@recetox.muni.cz; 2 Department of Pediatrics and Human Development, Institute for Integrative Toxicology, Michigan State University, East Lansing, MI 48824, USA; upham@msu.edu (B.L.U.); trosko@msu.edu (J.E.T.)

**Keywords:** carcinogenesis, carcinogens, gap junction intercellular communication, scrape loading-dye transfer

## Abstract

Dysregulation of gap junction intercellular communication (GJIC) is recognized as one of the key hallmarks for identifying non-genotoxic carcinogens (NGTxC). Currently, there is a demand for in vitro assays addressing the gap junction hallmark, which would have the potential to eventually become an integral part of an integrated approach to the testing and assessment (IATA) of NGTxC. The scrape loading-dye transfer (SL-DT) technique is a simple assay for the functional evaluation of GJIC in various in vitro cultured mammalian cells and represents an interesting candidate assay. Out of the various techniques for evaluating GJIC, the SL-DT assay has been used frequently to assess the effects of various chemicals on GJIC in toxicological and tumor promotion research. In this review, we systematically searched the existing literature to gather papers assessing GJIC using the SL-DT assay in a rat liver epithelial cell line, WB-F344, after treating with chemicals, especially environmental and food toxicants, drugs, reproductive-, cardio- and neuro-toxicants and chemical tumor promoters. We discuss findings derived from the SL-DT assay with the known knowledge about the tumor-promoting activity and carcinogenicity of the assessed chemicals to evaluate the predictive capacity of the SL-DT assay in terms of its sensitivity, specificity and accuracy for identifying carcinogens. These data represent important information with respect to the applicability of the SL-DT assay for the testing of NGTxC within the IATA framework.

## 1. Introduction

*“With respect to cancer causation, integration of the analyses suggest that the inhibition of gap junctional intercellular communication is involved in non-genotoxic cancer induction or in the non-genotoxic phase of the carcinogenic process (such as inflammation, cell toxicity, cell proliferation, inhibition of cell differentiation, and apoptosis)” [1].*

*“Here, we review the literature surrounding connexins in cancer cells in terms of specific connexin functions and propose that connexins function up stream of most, if not all, of the hallmarks of cancer” [2].*

These two compelling quotes [1,2], separated in time by nearly two decades of extensive research in the field of cancer, nicely sum up the motivation and rationale for this review paper. Here, we systematically searched currently available data on the ability of chemical substances to disrupt gap junctional intercellular communication (GJIC), as they were acquired by one of the most frequently used in vitro assays for this purpose, i.e., the scrape-loading-dye transfer (SL-DT) technique. The aggregated data on 328 individual chemicals that were published across nearly four decades of toxicological and biomedical research of GJIC are presented and discussed with respect to the utility of GJIC evaluation, specifically by the SL-DT assay, within the current framework for non-genotoxic carcinogen/carcinogenicity (NGTxC) assessment, which was recently endorsed by the OECD expert panel [3]. 

Cancer has emerged as a significant public health concern, currently representing the second most common cause of death among non-communicable diseases, after cardiovascular diseases, being responsible in 2020 for 19 million new health cases and 9 million deaths [4]. The cancer incidence is projected to further increase due to many factors [5]. Occupational or environmental exposures to carcinogenic pollutants have been recognized as important factors contributing to the development of cancers, with the incidence of cancer attributable to exposures to toxic chemicals estimated to be between 1 and 19% according to different studies (reviewed by [5]). Hence, there is a well-recognized need and effort to systematically identify and characterize cancer hazards of chemicals and assess the safety of their exposures to inform risk management to reduce cancer risks and ensure the protection of human health [5,6,7].

The issue of exposure to environmental carcinogens is of increasing societal and public health importance, particularly with respect to not only growing trends in global cancer incidence and some cancer-confounding factors (e.g., population aging) but also with the perspective of increasing global trends of chemical production [5], including novel compounds that might need to undergo carcinogenicity hazard identification, characterization and safety assessment [6].

Carcinogenesis is a multi-stage multi-mechanism process, which is generally considered to comprise three major operational stages: tumor initiation, promotion and progression [7,8,9,10]. The tumor initiation step involves mutation or alteration of genes, such as activation of oncogenes or inactivation of tumor suppressor genes, controlling cellular proliferation, survival, differentiation or DNA repair processes. The initiation step is assumed to occur primarily via a genetic change, e.g., due to oncoviruses, physical or chemical mutagens or genotoxicants. The promotion stage represents the lengthy, reversible and rate-limiting step of cancer, involving non-genotoxic or epigenetic alterations of signaling pathways and gene expression, leading to disruption of tissue homeostasis and clonal expansion of the initiated cell. Finally, progression represents the final stage of carcinogenesis, where further genetic and epigenetic changes occur in the promoted cells through genotoxic and non-genotoxic mechanisms, leading to the acquisition of the characteristic traits or ‘hallmarks’ of malignant cancer cells. The initially recognized six ‘hallmarks of cancer’ included unlimited growth, self-sufficiency in growth signals, insensitivity to anti-growth signals, apoptosis evasion, angiogenesis, the ability for tissue invasion and metastases [11]. Subsequently, additional cancer hallmarks have been proposed and discussed [7,12,13,14,15].

Chemical carcinogens can be classified into three main groups [16,17]: (1) ultimate carcinogens (chemicals with a direct action with the capacity to induce cancer without a previous metabolic activation), (2) procarcinogens (chemicals that need to be activated by metabolic activation to become ultimate carcinogens) and (3) co-carcinogens (chemical substances that cannot induce cancer when administered alone but can enhance the carcinogenic effect of other substances). From a toxicological and regulatory perspective, chemical carcinogens can be classified according to their prevailing mechanism as genotoxic carcinogens (GTxC), which include mutagenic or genotoxic agents inducing mutations and DNA damage by “errors of DNA repair” during initiation and eventually also progression stage. In contrast, NGTxCs (i.e., non-genotoxic carcinogens) represent agents whose carcinogenic activity does not depend on DNA damage but on various mechanisms altering cellular behavior during tumor promotion and progression stage [9]. In addition to an initiating agent being mutagenic, while a promoting agent is not mutagenic, there are other differences between the action of GTxC versus NGTxC. An initiating agent after repeated exposure in a small dosage or a single large exposure leads to carcinogenesis, in contrast to a promoting agent, which is not carcinogenic alone or when not exceeding a “threshold” limit. The duration and regularity of exposure rather than its intensity appear to be the most critical factors, as well as the absence of “anti-promotors”. An effect of an initiating carcinogen is irreversible and additive, whereas an effect of a promoting agent is reversible at the early stages [9].

These fundamental and traditionally recognized differences have been reflected in the testing and safety assessment approaches for the two groups of carcinogens. The rodent cancer bioassay is being challenged from the perspective of the 3Rs principle and regarding its utility and (in)ability to predict carcinogenicity in humans reliably [15,18,19,20,21]. The alternative, using in vitro testing methods and batteries, has already been established for GTxC, and some assays developed into OECD Test Guidelines [22]. Still, there are no available in vitro test guidelines addressing specifically human-relevant NGTxC [3]. To address the current lack of alternative testing tools and approaches, an OECD expert group developed an integrated approach to the testing and assessment (IATA) of chemical NGTxC [3,7]. Refined and structured in accordance with recognized cancer hallmarks and mechanistic knowledge, this IATA identified 13 key cancer hallmarks of NGTxC: (1) receptor binding and activation, including also hormone-mediated processes, and CYP P450 induction, (2) cell proliferation and (3) transformation, (4) GJIC (i.e., gap junction intercellular communication), (5) oxidative stress induction, (6) immunosuppression/immune evasion, (7) gene expression and cell signaling pathways, (8) increased resistance to apoptotic cell death, (9) pathogenic angiogenesis and neoangiogenesis, (10) genetic instability, (11) cellular senescence/telomerase, (12) invasion and metastasis and (13) epigenetic mechanisms [3,7]. These hallmarks are related to the key events occurring in the early to mid to later stages of the carcinogenic process. Based on this IATA framework and following the proposed assay evaluation criteria [3], appropriate tests, primarily in vitro assays, shall be identified and prioritized for further development and (pre)validation. The selected assay(s) will be targeted for validation needed for test guidelines and regulatory use. The representative standardized or commonly used tests (if available) addressing the key cancer hallmarks have recently been summarized, including the current status regarding their use in hazard assessment, availability of the test guidelines and their readiness level and eventually their inclusion into the OECD Test Guidelines Programme [3]. Cell-to-cell communication mediated through gap junction channels, i.e., GJIC, represents one of these essential key mechanisms for which there are currently no test guidelines or standardized tests [3]. 

GJIC is a fundamental biological cellular process in multi-cellular metazoan organisms that allows an exchange of various soluble ions and aqueous molecules between adjacent cells, allowing them to integrate multiple signals and coordinate their behavior in the tissues [23,24]. GJIC is a key mechanism for maintaining tissue homeostasis, and its dysregulation has been long recognized as a hallmark of NGTxC [2,3,7,14,24,25]. The inclusion of GJIC into the IATA of chemical NGTxC [3] has, thus, provided an incentive for evaluation, prioritization and further development of in vitro assays capable of addressing this specific hallmark, particularly with respect to the lack of existing test guidelines or candidate assays for GJIC hazard assessment within the OECD Test Guidelines Programme. 

Among various techniques developed for in vitro assessment of GJIC, the SL-DT (i.e., scrape loading-dye transfer) assay has probably been most frequently used in multiple studies of toxicant or carcinogen effects on GJIC. This in vitro assay is applicable to various cell types and cell lines. However, most of the published data focusing on the chemical effects on GJIC were generated using a rat liver epithelial cell line WB-F344. Nevertheless, such information, which would be highly relevant for further prioritization of in vitro assays suitable to address the GJIC hallmark in the IATA for NGTxC, has yet to be systematically mapped and summarized. Therefore, this review provides a brief overview of (1) the role of GJIC in maintaining tissue homeostasis and biological-mechanistic links to cancer/tumor promotion, (2) cell lines and methods suitable for in vitro GJIC assessment and, finally, and (3) the results of a systematic search of the application of the SL-DT assay to evaluate GJIC after the exposure to chemicals in a WB-F344 cell line. These in vitro data obtained from the systematic search are compared to IARC, CompTox/ToxRefDB and Oncologic classification of carcinogens, and the results (i.e., the SL-DT assay sensitivity, specificity and accuracy) are then discussed concerning the assay utility and its eventual further development for identification, characterization and safety assessment of NGTxC. 

## 2. GJIC as the Key Mechanism in Tissue Homeostasis

**Figure 1:**
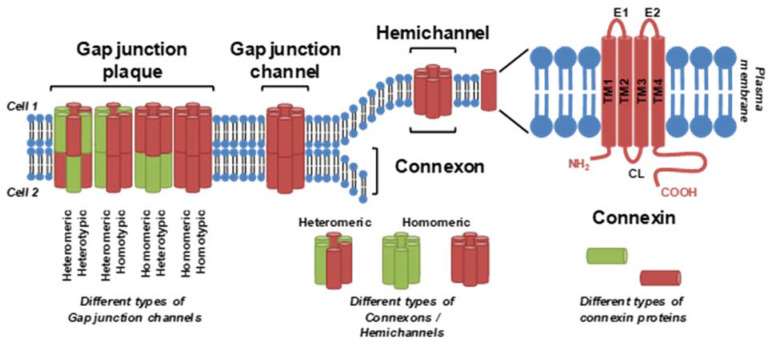
Connexins, connexin hemichannels and gap junction channels. A connexin monomer consists of an intracellular N-terminus, C-terminus and cytoplasmic loop (CL); four transmembrane segments (TM1-4); and two extracellular loops (E1 and E2). Six connexin subunits form a connexon or hemichannel, two connexons/hemichannels form a gap junction channel and multiple channels are clustered into a gap junctional plaque. Different types of connexin can be combined within one hemichannel, channel or plaque. From [27] and according to information from [23,25].

GJIC is facilitated by gap junctions, plaque-like protein structures that form contiguous channels between the cells. Vertebrate gap junctions are built from connexins (Cxs), which are membrane proteins with a tetraspan topology of four interspersed transmembrane domains connecting the cytoplasmic N-terminal region through an extracellular (E1), cytoplasmic and another extracellular (E2) loop to the C-terminal part of the Cx molecule [23,26] (Figure 1). This structure is shared among the 20 rodent or 21 human Cx species encoded by the family of *Gj/GJ* genes. In addition to the gene names, a nomenclature of Cxs based on the molecular weight predicted by DNA sequencing is also commonly used. For example, Cx43 denotes connexins with a predicted molecular weight of 43 kDa, encoded by rodent/human genes *Gja1*/*GJA1* [23]. In gap junction channels, six Cx protein units are organized into a hexameric hemichannel structure termed connexon.

A full gap junction channel is then formed by head-to-head docking of connexons from two adjacent cells. Expression of different Cx proteins is tissue- and cell type-specific, and different Cx species can be combined within a gap junction channel: homomeric connexons contain only one type of Cx, whereas heteromeric connexons are built from different Cx species [23]. Homotypic channels consist of two identical homomeric or heteromeric connexons, whereas heterotypic channels are created from two different homomeric or heteromeric connexons (Figure 1). This structure provides an opportunity to assemble various channels, e.g., with different permeabilities or regulations, although their functional meaning is still far from being completely understood [24]. Multiple (tens to thousands) Cx channels usually aggregate and create a gap junction plaque or a gap junction [26]. GJIC allows transferring of soluble ions and low molecular weight molecules (<1.2 kDa), including calcium ions, nutrients (e.g., glucose), amino acids (e.g., glutamate), nucleotides (e.g., ATP and ADP) and polyamines, but also secondary messengers and/or regulatory molecules, such as cAMP, cGMP, IP3 (inositol 1,4,5-trisphosphate), glutathione, miRNA or possibly small peptides [23,24,25,26]. In addition to forming gap junction channels and facilitating GJIC, Cxs have also been found to exhibit various non-coupling, GJIC-independent functions, and non-docked connexons are also known to function as hemichannels that allow the exchange of molecules, such as ATP and prostaglandins, between the cytoplasm and extracellular environment [24,25]. Moreover, pannexin proteins are related to invertebrate gap junction proteins, innexins, but share a similar topology with Cxs and form non-docked membrane channels in vertebrate cells, allowing communication between cellular cytosol and extracellular compartments [24,25]. 

**Figure 2:**
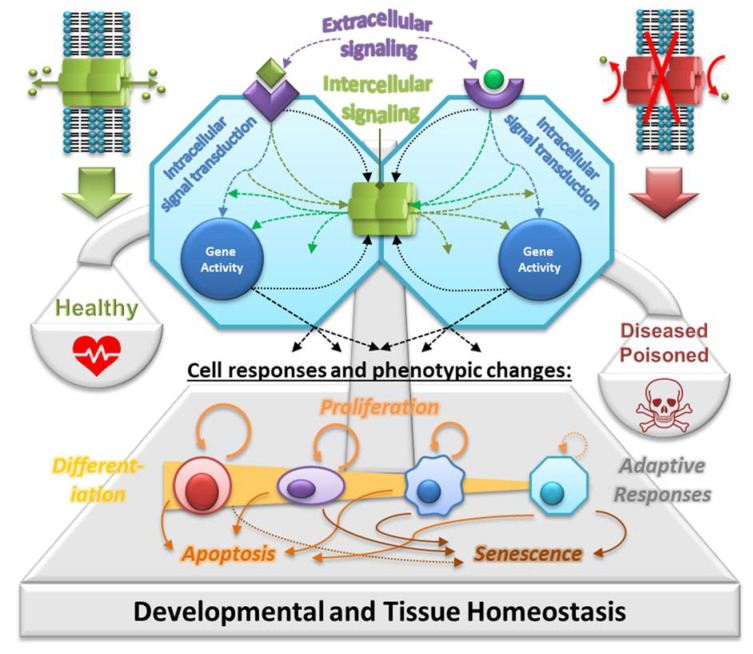
Gap junctions in homeostasis. Extracellular signals, such as growth factors, cytokines, hormones, toxicants, extracellular matrices and cell adhesion molecules, interact with receptor-dependent or receptor-independent targets, activating intracellular signal transduction pathways that induce gene transcription through activated transcription factors. These signals vary for each cell type: embryonic, adult stem, progenitor and terminally differentiated cells. Furthermore, these specific intracellular pathways operate under cascading systems that cross-communicate with each other in controlling the expression of genes that direct the proliferation, differentiation and apoptosis of cells within a tissue. These multiple intracellular signaling checkpoints are further modulated by intercellular signals traversing gap junctions—so-called gap junctional intercellular communication (GJIC). GJIC is crucial for integrating different signals and signaling mechanisms across the tissue, thus maintaining its homeostatic state under physiological conditions in a healthy organism. Abnormal interruption of the integrating signaling mechanism of GJIC by food-related and environmental toxins/toxicants will disrupt the normal homeostatic control of cell behavior. It can lead to an adverse outcome or a disease due to imbalanced proliferation, differentiation and/or apoptosis, which is typically observed, for example, in cancers and their tumor promotion and progression stage. Prepared according to information from [27,30,31].

GJIC represents a universal and key function of all Cxs. Among the neighboring cells in the tissue, GJIC allows integration of various signals and signaling mechanisms, such as extracellular signals (e.g., hormones, cytokines and growth factors) transduced by various secondary messengers and signal transduction pathways (e.g., IP3, cAMP and kinases), as well as other regulatory molecules and metabolites produced intracellularly during cell responses to different stimuli, microenvironment or various conditions (cell–cell and cell–extracellular matrix interactions, nutrients, catabolites, pH and temperature) (Figure 2). In this way, GJIC plays a central role in integrating signaling mechanisms controlling gene expression and coordinating cell behavior across the solid tissues of a multicellular organism, where gap junctions join virtually all differentiated cells, except free-flowing cells [28]. 

In fact, direct symplastic connections between adjacent cells in the tissues is an essential mechanism of cell communication in multicellular organisms with differentiated tissues and organs and possibly a prerequisite for their evolution and existence [29]. GJIC is the main mechanism of direct cell-to-cell coupling and intercellular communication in vertebrates, where it plays a key role throughout the entire ontogenic development. The development and function of a multicellular organism require tissue homeostasis, i.e., maintenance of internal steady state of organized populations of cell networks in a tissue, which involves removal of aged, damaged or developmentally no-longer-needed cells, and their renewal or replacement by new cells or cell types [27,30,31]. In this respect, four major cell types can be distinguished in the tissues according to their (a) potential for self-renewal and (b) potency, i.e., ability to differentiate into more specialized cell types: (i) totipotent and pluripotent stem cells, which occur typically only during the earliest stages of ontogenetic development, and their more differentiated progeny of (ii) multipotent, oligopotent or bipolar somatic (adult, tissue-specific or tissue-resident) stem cells, (iii) the progenitor, unipotent, or transiently-amplifying cells with a finite life span, and (iv) the terminally differentiated, nonproliferating cells. Maintenance of this homeostatic balance requires dynamic control of self-renewal, differentiation, proliferation and apoptosis of these different cell types, achieved by integrating growth-, differentiation- or apoptosis-inducing/inhibiting signals and conditions across the neighboring cells the tissue. Therefore, GJIC-dependent integration of various extra-, intra- and inter-cellular signals across the cells within a tissue is a key component of the systems control of cellular events that allows coordinating cell metabolism, gene expression and cell behavior between contiguous syncytium of cells organized into a hierarchal multicellular system (Figure 2). Thus, this mechanism is critical for maintaining tissue homeostasis via balanced cell proliferation, differentiation and apoptosis within a tissue [27,28,30,31,32]. 

Different levels of signal integration and intercellular communication are required during various developmental, physiological or cellular processes. Therefore, GJIC needs to be a tightly controlled process. GJIC can be regulated at different levels by various mechanisms: (a) control of Cx gene expression (i.e., transcription and translation of Cx genes), (b) Cx trafficking and turnover, which involves various posttranslational modifications controlling Cx maturation, connexon assembly, membrane localization and docking, as well as sequestration of Cxs from gap junction plaques and their degradation, and, finally, (c) channel gating mechanisms. Gating mechanisms allow rapid changes in the permeability of gap junction channels, presumably by conformational and structural changes. These can be controlled, e.g., by changes in voltage, calcium concentration, pH, redox balance and interactions between Cxs and other cellular proteins, such as kinases catalyzing the phosphorylation of Cxs at specific phospho-sites, or by interactions of Cxs with cytoskeleton or other membrane proteins [33]. Thus, different types of cellular stress, disruption of various cellular functions or perturbations of varying signal transduction pathways can lead to untimely inhibition or dysregulation of GJIC.

Impaired or dysregulated GJIC has been identified or implicated in the etiology of multiple diseases and pathologies [34,35]. With respect to its central signal integrating and tissue homeostatic function, GJIC dysregulation/untimely inhibition in normal cells has been connected to diseases involving disruption of tissue homeostasis, e.g., mitogenic signaling and proliferation, such as tumor-promoting stage of cancer and mechanisms of NGTxC [2,3,7,24,25]. Cancer was one of the first pathologies associated with gap junction channel impairment. All cancers can be generally viewed as disorders of tissue homeostasis when the cancer cells are characterized by dysregulation of growth (loss of contact inhibition, self-sufficiency in growth signals and insensitivity to growth-inhibitory signals), evasion of apoptosis and inability to terminally differentiate in combination with acquisition of phenotypic traits allowing to invade and metastasize in the other parts of the body [11,12,13,14]. (Dys)regulation of these cellular and tissue processes depends on various signaling mechanisms, including GJIC as a critical mechanism of signal integration in the tissues, as was recently and thoroughly reviewed and exemplified [2,24,25,35,36,37,38]. Thus, the lack of GJIC, its disruption or untimely dysregulation, e.g., by exposures to tumor-promoting factors or NGTxC like the prototypical tumor promotor TPA (12-O-tetra-decanoylphorbol-13-acetate) or the pesticide lindane [39], seems to be necessary for the cell to escape normal tissue homeostatic regulations and express or manifest the traits characteristic for malignant cancer cells, so-called hallmarks of cancer [2,14,24,36]. Inhibition of GJIC appears to be critical, especially for the early, rate-limiting tumor-promoting phase of cancer characterized by the expansion of the initiated cells [14,36].

The role of Cxs in cancer and carcinogenesis is very complex and context-dependent [2,24,25,28,31,35,36,37,38,40,41,42,43]. Most importantly, it depends on Cx type and isoform, cell and tissue type, types of interacting cells (among normal cells, among cancer cells and between normal and tumor cells), specific microenvironment, cancer stage or process (proliferation, apoptosis, metastasis and invasion, angiogenesis and epithelial-mesenchymal transition) and also type of Cx function (GJIC-dependent, non-junctional activity and Cx hemichannel activity). Cxs and GJIC can exhibit rather a tumor-suppressing activity in certain contexts, particularly during the tumor-promoting phase of cancer, while they can also facilitate specific tumor enhancing processes, e.g., during tumor progression and metastasis [2,24,25]. 

Nevertheless, there is substantial evidence associating the impairment of GJIC particularly with the tumor-promoting process in solid tissues. Here, typically, (1) exogenous and endogenous tumor promoters reversibly inhibit GJIC; (2) activation of oncogenes inhibits GJIC, and cancer cells exhibit reduced levels of GJIC; (3) tumor suppressor genes up-regulate GJIC; (4) anti-tumor promoters and chemopreventive agents up-regulate GJIC; (5) restoration of GJIC in tumorigenic cells via transfection with Cx genes at least partially restores normal growth and morphology of the cells and reduces their tumorigenicity; (6) antisense gap junction genes transfected into cancer cells augment foci formation; (7) Cx-knockout mice exhibit a higher rate of spontaneous or chemically or radiation initiated tumors [2,24,25,28,31,35,36,37,38,40,41,43]. Thus, loss of GJIC during the early stages of carcinogenesis and tumor onset is still considered an important hallmark, which could be utilized in screening in vitro methods for tumor-promoting/NGTxC activity or discovery of cancer chemopreventive drugs or dietary compounds [2,7,24,35,43]. However, that requires availability and accessibility of (a) suitable cell lines or in vitro cellular models with either basal (GJIC-competent cells) or inducible (GJIC-deficient cells) and measurable levels of GJIC, as well as (b) techniques for GJIC evaluation with acceptable operability and sufficient throughput.

## 3. Cell Lines and Methods for In Vitro GJIC Assessment

The level of GJIC can be effectively measured in vitro in different types of GJIC-competent or GJIC-defective (deficient) cell models, including primary cells, stem cells or permanent cell lines using a variety of methods [44,45]. 

Examples of primary cells used for functional assessment of GJIC include representatives of various organs, tissues and cell types isolated mostly from rodents (rat, mouse) and other animals (e.g., sheep, piglets) or humans. Most notably, GJIC has been assessed in cultured primary cells isolated from, e.g., the nervous system [46,47,48,49,50], liver [45,51], intestine [52], kidney [53,54,55,56], lung [57,58], smooth muscles, including myometrial cell cultures [59,60,61], cardiac myocytes [62,63], ovaries [64,65,66,67], prostate cells [68] or testicular cells [63,67,69,70,71].

However, permanent cell lines are more suitable than primary cells for in vitro cell-based assays for routine toxicity assessments if a specific molecular target or process of interest is expressed or present [72]. They are also more suited to allow standardization and higher throughputs. An overview of established mammalian cell lines, commonly utilized for GJIC assessment, is provided in Table 1, along with their identifiers, major Cx types detected in these cells and methods used for Cx detection and GJIC evaluation. These cell lines include representatives of various tissues and organs (e.g., brain, liver, intestine, kidney and skin) isolated from rodents or humans. The most frequently used cell lines are rat liver epithelial cell lines such as WB-F344, IAR-20 or Clone 9. The major studied Cx in mammalian cell lines in connection with functional assessment of GJIC has been Cx43, followed by Cx26, Cx32 or Cx45, as also reported previously [44]. Cx43 represents a Cx isoform expressed in most tissues and cell types, particularly abundant in epithelial cells, where it is often the main component of gap junctions [73]. Since over 90% of human cancers account for carcinomas, i.e., solid tumors derived from epithelial cells [39], Cx43 has been the most explored Cx type in carcinogenesis [35]. Expression of Cx43, either mRNA or protein, is a clinically relevant marker for some cancer types, including colorectal, bladder, lung or liver cancers, bone metastases, glioma or melanoma [41,74]. In the liver, Cx43 is predominantly expressed in nonparenchymal liver cells and hepatocyte precursors, whereas differentiated parenchymal hepatocytes harbor Cx32 and Cx26 [74]. All these types of connexins are associated with hepatocellular carcinoma (HCC) development [74].

Several signal transduction pathways controlling GJIC have been identified in vitro and include mitogen-activated protein kinase (ERK1/2, p38) [75,76,77,78,79,80,81,82,83], protein kinase C [77,80,81,82,84,85,86,87,88], protein kinase A [82,89,90], phosphatidyl choline specific phospholipase C [78,89,90], diacylglycerol lipase [89,90], calcium-independent phospholipase 2 [89] and Src [82,90,91,92,93,94,95]. Knowing which signal transduction pathways are involved in NGTxC-induced dysregulation of GJIC will be important in assessing the potential carcinogenicity of individual chemicals and their mixtures. For example, most polycyclic aromatic hydrocarbons (PAHs) disrupt GJIC through a phosphatidylcholine-specific phospholipase C mechanism. Thus, the effects of PAH mixtures would be predicted to be additive [96]. 

The assays suitable for evaluating GJIC have been extensively reviewed, including discussions on their principles, applicability, advantages and disadvantages [27,97,98,99]. These assays can be principally divided into three major groups based on the technical approaches used for estimating GJIC capacity. Namely, there are assays based on the measurements of (a) electrical conductance (electrical coupling), such as the double whole-cell voltage-clamp (DWCV) technique, (b) endogenous metabolite transfer (metabolic cooperation assays, MCs) or (c) a fluorescent dye transfer (DT). The latter group involves a variety of techniques, such as fluorescence recovery after photobleaching (FRAP), local activation of fluorescent molecular probe (LAMP), microinjection (MI), scrape loading (SL) or preloading (Pre) and parachute (Par) assays. 

**Table 1:** Overview of cell lines commonly utilized for GJIC assessment with major studied connexins (Cx) and used methods.

Organ/Cell Line	Species	Major Connexins (Method)	GJIC (Method)	Ref.
Brain:				
BT5C1	R		Y (MI)	[100]
RG2	R	Cx43 (WB)	Y (SL)	[101]
RGC	R		Y (SL)	[27]
Ear:				
HEI-OC1	M	Cx26, Cx30, Cx31, Cx43 (WB, IF, RT-qPCR)	Y (SL)	[102]
Eye:				
RGC-5	R/M		Y (SL)	[103]
Intestine:				
Caco-2	H	Cx43, Cx26 (RT-PCR, WB, IF)	Y (SL)	[27,104,105,106]
IEC-6	R	Cx43 (RT-PCR, WB)	Y (MI)	[107]
Kidney:				
BHK 21/13	GH		Y (MC, MI)	[108]
G401.2/6TG.1	H		Y	[109]
MDCK	D	Cx43 (WB)	Y (SL)	[110]
Liver:				
ARL-18	R	Cx43 (WB, IF)	Y (FR)	[111]
BRL	R		Y (MI, MC)	[100,108]
BRL 3A	R	Cx43, Cx32 (RT-(q)PCR, IF, WB)	Y (SL, Par)	[112,113,114,115,116,117,118,119,120]
Chang Liver	H		Y (SL)	[121]
Clone 9	R	Cx43, Cx26 (WB, IF, RT-qPCR)	Y (SL, MI, FR)	[122,123,124,125,126,127,128,129,130,131]
G27	R		Y (MI)	[132,133,134]
HepG2	H	Cx43, Cx32 (RT-PCR)	Y (MI, Par, SL)	[121,135,136,137]
HL1-1	H		Y (SL)	[27]
HLEC-04	H		Y (MI)	[138]
Huh-7	H		Y (SL)	[121]
IAR-20	R	Cx43 (RT-PCR, WB, IF)	Y (SL, MI, Par, MC)	[139,140,141,142,143,144,145,146,147,148,149,150,151,152]
IAR-203	R	Cx43 (WB, IF, RT-PCR)	Y (MI)	[153,154]
IAR-6.1	R	Cx43 (WB)	Y (SL)	[81,141,143,144]
N1S1-67	R	Cx43 (WB, NB)	Y (Pre)	[155]
REL	R	Cx43 (WB, IF)	Y (MI)	[156,157,158,159]
T51B	R	Cx43 (WB, NB)	Y (MI)	[160,161,162,163,164]
WB-F344	R	Cx43 (WB, IF, RT-(q)PCR)	Y (SL, MI, Par, DWCPC, MC, FR, Pre)	[39,51,75,78,79,89,90,103,165,166,167,168,169,170,171,172,173,174,175,176,177,178,179,180,181,182,183,184,185,186,187,188,189,190,191,192,193,194,195,196,197,198,199,200,201,202,203,204,205,206,207,208,209,210,211,212,213,214,215,216,217,218,219,220,221,222,223,224,225,226,227,228,229,230,231,232,233,234,235,236,237,238,239,240,241,242,243,244,245,246,247,248,249,250,251,252,253,254,255,256]
Lung:				
16HBE14o-	H	Cx43 (IF)		[257]
A549	H	Cx43 (IF)		[258]
Beas-2B	H		Y (SL)	[27,259]
C10	M	Cx43 (IF, WB)	Y (MI)	[27,260]
HBE1	H	Cx43 (WB)	Y (SL)	[259,261]
V79	CH		Y (MC, MI)	[108,236,262,263,264,265,266,267]
Mammary gland:				
BICR/M1Rk	R	Cx43 (WB, RT-PCR)	Y (MI, SL)	[100,268]
Pancreas:				
H6c7	H	Cx43 (WB, IF, RTPCR)	Y (SL)	[269]
Placenta:				
FL	H		Y (MI)	[100]
Prostate:				
RWPE-1	H	Cx43, Cx32 (WB)	Y (SL, FR)	[270,271]
Skin:				
3PC	M		Y (MI)	[136]
3T3	M		Y (MI, MC)	[108,272]
CA3/7	M		Y (MI)	[136]
HaCaT	H	Cx43, Cx26 (RT-PCR, WB, IF)	Y (FR, MI, SL)	[273,274,275]
HEL37	M		Y (MI)	[100]
Testes:				
42GPA9	R	Cx43 (WB, RT-PCR, IF)	Y (FR)	[276]
LC540	R		Y (FR, SL)	[236]
SerW3	R	Cx43 (RT-qPCR, IF)	Y (MI)	[277]
TM3	M	Cx43, Cx45 (WB, RT-PCR, IF)	Y (SL)	[77,278]
TM4	M	Cx43, Cx45 (WB, RT-PCR)	Y (SL, MI)	[77,279]

Abbreviations: Methods: **DWCPC**, dual whole-cell patch-clamp; **F**, gap-FRAP (gap fluorescence recovery after photobleaching); **IF**, immunofluorescence; **MC**, metabolic cooperation; **MI**, microinjection; **Par**, parachute assay; **Pre**, preloading assay; **NB**, northern blotting; **RT-(q)PCR**, reverse-transcription-(quantitative) polymerase chain reaction; **SL**, scrape loading; **WB**, western blotting; Species: **CH**, Chinese hamster; **D**, dog; **GH**, golden hamster; **H**, human; **M**, mouse; **R**, rat; Cell lines: **16HBE14o-**, human bronchial epithelial cell line (RRID:CVCL_0112; Sigma-Aldrich #SCC150); **42GPA9**, murine Sertoli cell line (RRID:CVCL_U464); **3PC**, mouse epidermal initiated cells (RRID:CVCL_JW71); **3T3**, mouse embryonic fibroblasts (ATCC CRL-1658™); **A549**, adenocarcinomic human alveolar basal epithelial cell line (ATCC CCL-185™); **ARL-18**, adult rat liver epithelial-like cell line (RRID:CVCL_4Z20); **Beas-2B**, human bronchial epithelial cell line (ATCC CRL-9609); **BHK 21/13**, golgen hamster normal embryonic kidney fibroblasts (ATCC CCL-10™); **BICR/M1Rk**, fibroblastoid cells derived from a rat mammary tumor (RRID:CVCL_4128); **BRL**, buffalo rat liver cell line (RRID:CVCL_4565); **BRL 3A**, buffalo rat liver cell line 3A (ATCC CRL-1442™); **BT5C1**, rat glioma cells; **CA3/7**, mouse epidermal carcinoma-derived cell line (RRID:CVCL_JW73); **Caco-2**, human epithelial colorectal adenocarcinoma cells (ATCC HTB-37™); **C10**, murine pulmonary epithelial cell line; **Chang Liver**, Human papillomavirus-related endocervical adenocarcinoma (a HeLa derivative, ATCC CCL-13™); **Clone 9**, normal rat liver epithelial cell line (ATCC CRL-1439™); **FL**, epithelial cells derived from human amniotic membrane (a HeLa derivative, RRID:CVCL_1905); **G27**, rat hepatoma cell line; **G401.2/6TG.1**, human kidney epithelial cell line; **H6c7**, human pancreatic ductal epithelial cell line (RRID:CVCL_0P38); **HaCaT**, aneuploid immortal keratinocyte cell line from adult human skin (RRID:CVCL_0038); **HBE1**, immortalized human bronchial epithelial cell line (RRID:CVCL_0287; Kerafast #ENC002); **HEI-OC1**, conditionally immortalized mice cochlear cells (RRID:CVCL_D899); **HEL37**, mouse epidermal cells (RRID:CVCL_6D73); **HepG2**, human liver cancer cell line (ATCC HB-8065™); **HL1-1**, adult human liver stem cells; **HLEC-04**, human hepatocyte line derived from SV40 T antigen transfected primary cultured human hepatocytes; **Huh-7**, adult human hepatocellular carcinoma cell line (RRID:CVCL_0336); **IEC-6**, rat normal intestinal epithelioid cell line (ATCC CRL-1592™); **IAR-20**, non-transformed rat liver epithelial cells (RRID:CVCL_5296); **IAR-203**, non-transformed rat liver epithelial cells; **IAR-6.1**, non-transformed rat liver epithelial cells (RRID:CVCL_D613); **LC540**, rat adult Leydig cell adenoma cell line (ATCC CCL-43™); **MDCK**, Madin Darby Canine Kidney (ATCC CCL-34™); **N1S1-67**, rat hepatoma cell line; **REL**, rat liver epithelial cell line; **RG2**, rat glioma cells (ATCC CRL-2433™); **RGC**, rat glial cells; **RGC-5**, rat/mouse retinal ganglion cell line (RRID:CVCL_4059); **RWPE-1**, human prostate epithelial cells (ATCC CRL-11609™); **T51B**, rat liver nonparenchymal cell line; **TM3**, murine immortalized immature Leydig cell line (ATCC CRL-1714^TM^); **TM4**, murine immortalized immature Sertoli cell line (ATCC CRL-1715^TM^); **V79**, Chinese hamster lung fibroblasts (RRID:CVCL_2234; ECACC 86041102); **WB F344**, normal rat liver epithelial cell line (RRID:CVCL_9806; JCRB0193). Others: **Y**, yes.

One of the important drawbacks for most of the techniques traditionally used for GJIC evaluation is their limited throughput and sometimes a requirement for special equipment or skills. However, some of these methods have been recently adapted into formats compatible with a high throughput screening (HTS) and/or high content analysis (HCA)/high content screening (HCS). These adapted methods, with their advantages or disadvantages, are summarized in Table 2 (modified and updated from [259]). Some HTS and HCA/HCS techniques rely on a fluorometric or luminometric sensing of specific molecules exchanged via gap junctions composed by Cx43 between donor and recipient cells, i.e., metabolic cooperation. However, most of these setups are based on dye-transfer techniques, such as MI, Par/Pre, microfluidic loading, electroporation loading (EL-DT) or laser perforation (LP-DT). They also include the SL-DT assay, probably the most frequently used assay to study GJIC in the context of toxicology and toxicant-induced tumor promotion.

## 4. Scrape Loading-Dye Transfer Using the WB-F344 Cell Line

**Figure 3:**
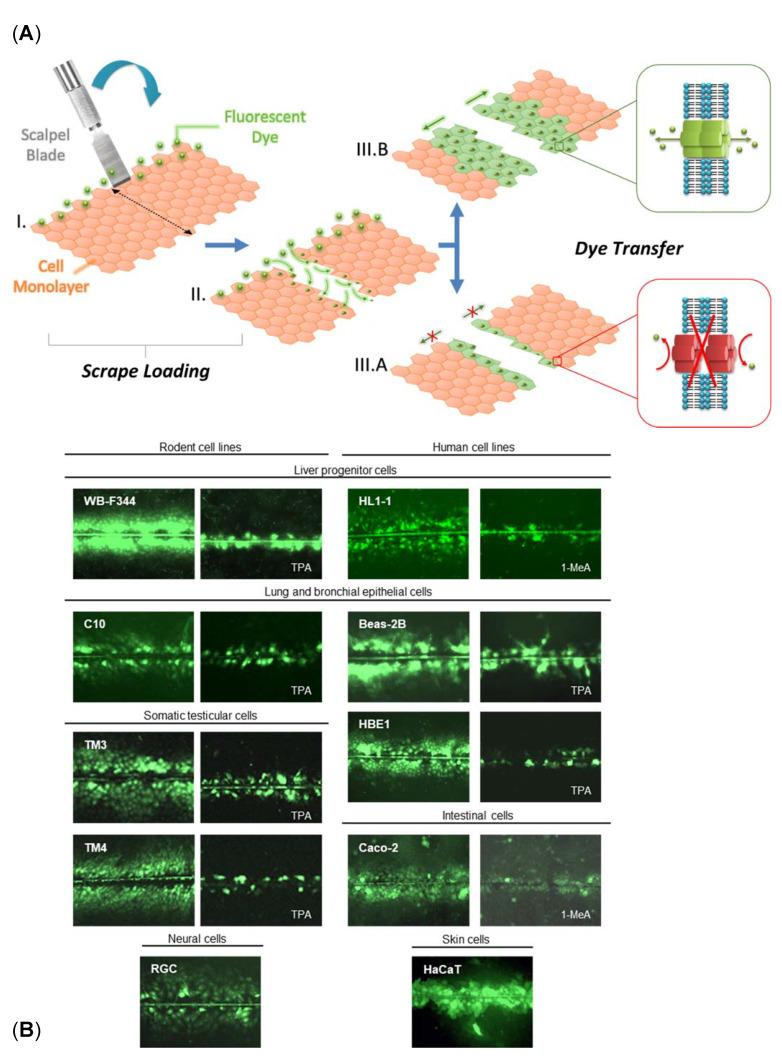
GJIC documented by the SL-DT technique in different cell lines. (**A**) Principle of the SL-DT assay. Fluorescent gap junction permeable dye (e.g., Lucifer Yellow) is loaded into the cell monolayer by a clean cut with a sharp scalpel blade (I–II). Subsequent dye transfer through the cell monolayer depends on the number of gap junctions and their functional state, and it is proportional to the levels of GJIC (IIIA-B). (**B**) Illustrative microphotographs of different permanent cell lines where the levels of GJIC were evaluated by the SL-DT technique, either in nontreated cells or following treatment with a model GJIC inhibitor, such as 12-O-tetra-decanoylphorbol-13-acetate (TPA) or 1-methylanthracene (1-MeA). **Beas-2B**, human bronchial epithelial cell line (ATCC CRL-9609); **Caco-2**, human epithelial colorectal adenocarcinoma cells (ATCC HTB-37™); **C10**, murine pulmonary epithelial cell line; **HaCaT**, an aneuploid immortal keratinocyte cell line from adult human skin (RRID:CVCL_0038); **HBE1**, immortalized human bronchial epithelial cell line (RRID:CVCL_0287; Kerafast #ENC002); **HL1-1**, adult human liver stem cells; **RGC**, rat glial cells; **TM3**, murine immortalized immature Leydig cell line (ATCC CRL-1714^TM^); **TM4**, murine immortalized immature Sertoli cell line (ATCC CRL-1715^TM^); **WB-F344**, normal rat liver epithelial cell line (RRID:CVCL_9806; JCRB0193). Prepared according to information from [27].

The most commonly used technique for in vitro GJIC assessment is the SL-DT assay [97]. The original SL-DT assay was reported by [292]. This technique uses a membrane-impermeable and gap junction-permeable fluorescent dye, typically Lucifer Yellow, introduced into adherent cells grown in a Petri dish by scraping with a rubber policeman or wooden probe [292]. The relatively invasive scraping step has been replaced by a clean cutting step with a sharp blade, such as a surgical scalpel blade. This later version was dubbed as the “scalpel loading-dye transfer” technique, the protocol was modified accordingly [33,293], and shown to be applicable to different types of cells (Figure 3). Nevertheless, the SL-DT assay has been recently further modified to increase the assay throughput and obtain more information from the GJIC assay by using multiple fluorophores and evaluating numerous parameters. The updated multiparametric SL-DT (mSL-DT) assay thus uses a standard microplate format and brightfield and fluorescence microscopic imaging of cellular staining done with a combination of three different fluorescent dyes (Lucifer Yellow LY for GJIC evaluation, Propidium Iodide for GJIC and viability evaluation, Hoechst 33342 for cell density evaluation) [259]. This setup allows assessing GJIC and additional parameters, such as cell density and viability, and applying HCA/HCS pipelines. This mSL-DT technique has also been used for various adherent cell types (Table 2) because its advantage is that no specialized cell model is needed. Both the SL-DT and mSL-DT can be documented using a standard widefield fluorescence microscope equipped with suitable Ex/Em filters and a digital camera. Additional specialized equipment, cell models or technical skills are not required. This method can eventually also be done ex vivo in the tissues of interest, such as liver tissue slices of rodents exposed ex vivo or in vivo [33,227].

Currently, the most extensively used cell line for GJIC characterization using the SL-DT assay is normal rat liver epithelial/oval cells WB-F344 cells isolated from Fischer F344 rats fed a choline-deficient, ethionine supplemented diet to enrich for oval cells. WB-344 cells represent probably one of the best-characterized rat liver epithelial/oval cell lines [294,295]. These cells express primarily gap junctional protein Cx43 and communicate via GJIC [296]. They are diploid, nontumorigenic and multipotent, with a proliferation capability of immortal cell lines. When transplanted into syngeneic Fischer F344 rats, they undergo morphological differentiation into hepatocytes, incorporate into hepatic plates or differentiate into biliary duct cells [297,298]. WB-F344 cells can also transdifferentiate into cardiac myocytes when transplanted into cardiac tissue [299].

WB-F344 cells have been frequently utilized for studying the carcinogenicity process, including chemically induced carcinogenicity. In vitro neoplastic transformation of WB-F344 cells was repeatedly demonstrated by (a) a chemically induced two-step (initiation/promotion) transformation procedure [300,301,302], (b) mutagenizing [303], (c) overexpression of various oncogenes [296,304,305,306] or (d) spontaneously upon chronic maintenance in a confluent state [307]. Transformed WB-344 cells typically become deficient in GJIC and tumorigenic in vivo [296,305,308,309]. On the other hand, the neoplastic phenotype of transformed WB cells was attenuated or reversed by chemopreventive agents stimulating GJIC [43] or by a forced expression of gap junctional proteins Cxs [309,310]. These findings indicate that these cells represent possible precursor cells in the development of liver cancers and provide evidence for the key role of GJIC and its dysregulation during their neoplastic transformation and tumorigenic process. It supports the biological plausibility of the evaluation of GJIC in this particular cell type in connection to NGTxC in the liver tissue. 

WB-F344 cells have been applied for studying tissue homeostasis and its disruption. Disruption of tissue homeostasis does not necessarily require a full closure of channels. Transfection of WB-F344 cells with a dominant-negative *Cx43* gene (DNCx43) resulted in decreased GJIC (>50%) when LY-649 (Lucifer Yellow with MW = 649) was used [311]. However, normal GJIC was observed when LY-457 (MW = 457) [311] was used, indicating that DNCx43 channels only allowed intercellular communication of low molecular weight (MW) (<650 Da) to effectively pass through gap junctions as compared to normal intercellular communication in the parent cell line, which allows intercellular communication of signals with molecular weights up to 1000 Da. Normal cell proliferation was observed in the DNCx43 cell line, but cell differentiation into biliary duct cells was blocked [311], indicating that high MW signals are involved in differentiation and partial closure of channels can also interrupt tissue homeostasis.

## 5. Chemical Effects on GJIC in WB-F344 Cells Evaluated by SL-DT Assay—A Systematic Search

### 5.1. Summary of the Results

We systematically searched the literature to gather chemicals assessed by the SL-DT-based assays in liver cells, specifically in WB-F344 cells. We found 86 papers reporting the GJIC activity of 328 chemicals from 64 different chemical classes (see Section 7 and the supplementary information for methodology, Supplementary Table S1 and File S1—a summary of the results reported from the SL-DT assays on 328 chemicals). The most studied compounds were polychlorinated biphenyls (48 PCBs) and their metabolites (38), followed by PAHs (37 PAHs) and their derivatives, metabolites or transformation products (39). Other compounds principally studied were phthalates (20), per- and polyfluoroalkyl substances (14 PFASs) and organochlorine compounds (11). The SL-DT assay classified 232 compounds as positive (i.e., inhibiting GJIC), 93 as negative (i.e., non-inhibiting GJIC) and 3 compounds with equivocal results. Chemicals that are categorized as positive (232 chemicals in total) can be divided into three categories: (1) the chemicals with the EC_50_ values in nM ranges, such as TPA (chemical No. 281 in Supplementary Table S1 and File S1), 2,3,7,8-tetrachlorodibenzo-p-dioxin (TCDD, No. 259), tumor necrosis factor-alpha (TNFα, No. 263) or epidermal growth factor (EGF, No. 261), (2) the chemicals with the EC_50_ values in 1–100 µM range, such as dichlorodiphenyltrichloroethane (DDT, No. 84) or methoxychlor (No. 88) and numerous low molecular weight PAHs, and (3) the chemicals that exhibit just weak GJIC-inhibitory activity, i.e., they were not able to cause 50% GJIC inhibition (i.e., EC_50_ values cannot be calculated). There were 38 such weak GJIC-inhibitory compounds, including, for example, di(2-ethylhexyl) phthalate (DEHP, No. 283) or benz[a]anthracene (No. 100). 

Most of the compounds were able to inhibit GJIC rapidly (within 1 h), suggesting that GJIC dysregulation by most of these chemicals occurred primarily by non-genomic mechanisms. That means that the effect was elicited by immediate modulations of signal transduction pathways (e.g., kinases and phospholipases) and other mechanisms controlling the gating of Cx channels and Cxs fate (localization, sequestration from gap junctional plaques and degradation), rather than by changes in gene expression induced by activated transcription factors [78,186,234]. In contrast, a few chemicals, specifically TCDD (No. 259), TNFα (No. 263) and 17β-estradiol (No. 323), did not affect GJIC after shorter exposure times but inhibited GJIC after the prolonged exposure (24 h). Additionally, several other compounds showed only weak effects after <1 h exposure, and their effects became stronger after 24 h (e.g., DEHP, No. 283; diisononyl phthalate, DINP, No. 293; dioctyl phthalate, DOP, No. 296). Apparently, other compounds can inhibit GJIC via mechanisms other than rapid dysregulation of gating. These mechanisms could involve, for example, changes in GJIC regulation depending on alterations of gene expression, e.g., due to transcription factors activated after chemical interaction with corresponding receptors (e.g., aryl hydrocarbon (AhR)-, TNF-, estrogen- or peroxisome proliferator-activated (PPAR)-receptors, respectively). Moreover, delayed inhibition of GJIC can also be caused by indirect mechanisms, when a chemical can induce an extracellular release of signaling molecules, such as inflammatory cytokines, which later cause modulation of GJIC. On the other hand, the rapid effects of some GJIC inhibitors following a single dose are transient, recovering from GJIC inhibition after several hours of exposure (e.g., TPA (No. 281), EGF (No. 261), vinclozolin (No. 94), lindane (No. 87), and diphenyl phthalate (DPhP, No. 298) [81,228,234,235,312]). Thus, both short-term (≤1 h) as well as longer (>12–24 h) exposure times should be included in the experimental design when assessing effects of chemicals on GJIC to detect and discriminate compounds disrupting GJIC rapidly but possibly transiently, and compounds inhibiting only after the prolonged exposures [33]. In addition to reducing the possibility of false-negative results, the knowledge on the kinetics of GJIC inhibition can indicate the character of the mechanism involved, i.e., rapid interference with Cx channels and their gating regulation, or rather their alterations through changes in Cx gene expression, which can be further deciphered in follow-up experiments. Furthermore, the ability of the cells to recover from GJIC inhibition and the kinetics of this process can also provide mechanistically and toxicologically important information. When tested in such a setup, most compounds inhibited GJIC reversibly, and GJIC was restored after washing out the chemical from the cell culture medium, as demonstrated, for example, for several low molecular weight PAHs [194], cannabinoids (cannabinol, No. 7, delta-9-tetrahydrocannabinol THC, No. 8) [79], organic peroxides (benzoyl peroxide, No. 76, dicumyl peroxide, No. 77) [184], methoxychlor (No. 88) or vinclozolin (No. 94) [235], PFASs (perfluorodecanoic acid PFDA, No. 268, perfluorooctane sulfonate PFOS, No. 274, perfluorooctanoic acid PFOA, No. 276) [172] or ceramides (C6 ceramide, No. 321, C8 ceramide, No. 322) [238]. The kinetics of the recovery can indicate possible mechanisms involved in GJIC inhibition when a rapid recovery can be expected as in the case of dysregulation of GJIC via channel gating. In contrast, longer recoveries would indicate GJIC inhibition caused by mechanisms interfering with Cx fate or gene expression. If there is no recovery of GJIC, then cytotoxicity and cellular damage might be a factor contributing to GJIC impairment and should be further assessed. If a compound does inhibit GJIC irreversibly, then the implications for the health of an organism could be quite different from most other agents and needs to be part of the hazard and risk calculations [33].

Importantly, the indirect mechanisms of GJIC inhibition might involve cells autocrinally (dys)regulating their GJIC through the production and release of extracellular signals and paracrine signaling from other cell types in the tissue affected by the chemical. Therefore, such complex mechanisms of disruption of tissue homeostatic control, which involve cell-specific effects and interactions of multiple cell types, shall also be considered and reflected in the eventual testing approach, especially for the correct interpretation of negative GJIC results. Critically important information could be obtained from the other assays in a NGTxC testing strategy, addressing other relevant key endpoints, such as immune and inflammatory responses.

### 5.2. Reproducibility of the Assay

In Supplementary Table S1, the retrospective interlaboratory repeatability and reproducibility of the SL-DT assay can be estimated from the studies testing the same chemicals. Out of 328 chemicals in the dataset, the effects on GJIC were reported by more than a single study for 52 compounds. The separate studies working with the same chemical observed mostly results and benchmark values (e.g., positivity or negativity, similar EC_50_ values or concentrations needed to induce nearly complete inhibition of GJIC, within comparable time frames) comparable to each other, which were (re-)produced independently in several labs. The widest range in the effective reported concentrations was found for a recognized tumor promoter, hydrogen peroxide (No. 265), with the values shown to inhibit GJIC ranging between <100 µM to over 1 mM according to 17 studies. However, in most of these studies, hydrogen peroxide was applied as a model compound only in a single dose to inhibit GJIC, which does not allow us to understand the concentration–response relationship clearly. Furthermore, low stability of the compound could also be contributing to a wider range of effective concentrations being used, as the results might depend on the specific ways of handling the compounds and possibly varying details in the experimental setup (e.g., source of the compound, its storage, diluting steps, delivering to the testing system and also cell culture medium composition). 

According to our search, another prototypical tumor promoter and potent GJIC inhibitor, TPA (No. 281), was the most tested compound, assessed in 22 studies using the SL-DT assay in WB-F334 cells. TPA dysregulated GJIC in all these studies with the EC_50_ value ranging from 0.002 to 0.02 µM [78,90,167,186,187,190,196,203,204,205,208,209,211,213,222,228,229,230,231,232,233,302]. This difference represents a relative difference of one order of magnitude but falls within a relatively narrow interval of 18 nM on the absolute scale. 

The next most frequently studied chemicals by the SL-DT assay in WB-F344 cells were fluoranthene (No. 124), with EC_50_ values ranging between 9 and <70 μM according to nine studies [78,166,177,186,193,194,196,199,200], and 1-methylanthracene (No. 140), with EC_50_ values between 11–40 µM as found in seven papers [78,89,192,193,194,195,235]. A relatively wider range of reported effective concentrations was also found in two studies conducted with arachidonic acid (No. 53) and another two papers with benzo[a]pyrene (No. 102), where the EC_50_ values were estimated to be between 5 and <70 μM for arachidonic acid or from 10 to 100 uM for benzo[a]pyrene. On the other hand, the reported effects of 40 other repeatedly studied chemicals appeared to be very uniform, with estimated EC_50_ values within the same order of magnitude and/or with a difference between the independently reported values being less than three-fold. The compounds reported in three or more reports include DDT (No. 84), lindane (No. 87), several PAHs (pyrene, No. 132, phenanthrene, No. 130, fluorene, No. 125), growth factors (EGF, No. 261), polychlorinated biphenyl PCB 153 (No. 208), pentachlorophenol (No. 90) or perfluorooctanoic acid (PFOA, No. 276).

Nevertheless, out of 52 chemicals investigated repeatedly for their effects on GJIC, 5 compounds provided equivocal results, i.e., they were reported by different studies as either GJIC-inhibiting or non-inhibiting compounds. However, anthracene (No. 99) and 2-methylanthracene (No. 146) were reported as GJIC-non-inhibiting compounds by the majority of studies. Anthracene was negative in four studies out of six [166,192,193,194,195,196], 2-methylanthracene in four out of five [89,192,193,194,195]. Thus, we considered these two compounds as negatives (Supplementary Table S1). Only three compounds, namely benzo[e]pyrene (No. 107) [166,196], dibenz[a,c]anthracene (No. 115) [196,198] and dibenz[a,j]anthracene (No. 117) [196,198], were found to be reported as GJIC-inhibitors or non-inhibitors in an equal number of studies, thus ranked as equivocal in Supplementary Table S1.

Such discrepancies in GJIC-inhibitory activity and variance of reported EC_50_ values could be attributed to different experimental setups and conditions, which can include (a) culture medium composition and serum content, (b) cell passages and seeding density, duration of the culture prior the exposure, (c) the compound (source, purity), solvent type and concentration, and also the method of exposing the cells (e.g., direct addition of the compounds to the medium, or partial/complete replacement of the medium), (d) the method of image analysis and evaluated parameters (area, distance, etc.) and means of data normalization. Unfortunately, based on the method description given in the included studies, it is difficult to identify the most important driver of repeatability and reproducibility of the SL-DT assay. The reporting and methodological qualities evaluated using SciRap for in vitro studies [313] are summarized in Supplementary File S2. The methodological quality was quite high for most studies. The average of the SciRap Methodology Index is 81% ranging from 25% to 100% (Supplementary File S2). However, the reporting quality of the included studies is a problem, specifically for old studies. The average of the SciRap Reporting Index is 61% ranging from 26% to 91% (Supplementary File S2). The most underreported area was the basics of the test system, including cell seeding density, the number of cell passages of the cell line used, the metabolic competence of the used system or the description of measures taken for avoiding or screening for contamination by mycoplasma, bacteria, fungi and virus. The second underrepresented area was the test compound and controls, covering mostly the purity and solubility of the test compound(s) or statement that an untreated and vehicle control were included. Problems with reproducibility can also be due to different approaches to analyzing the transfer of Lucifer Yellow dye. Within the included studies, the evaluated endpoint parameters were the distance/migration of Lucifer Yellow from scrape, the number of Lucifer Yellow-stained cells, the area of Lucifer Yellow-stained cells, the number of Lucifer Yellow-labeled cells in a row, or not specified. Additionally, different ways of data normalization were apparently used, including (a) presentation of not-normalized primary data, (b) data normalized to the negative, solvent or non-specified control or (c) different approaches to account for the cells loaded with the dye. Overall, interlaboratory repeatability and reproducibility of the SL-DT assay still remain to be parametrized and quantified by using a synchronized standard operation procedure (SOP). Nevertheless, we observed very good reproducibility of the SL-DT assay in WB-F344 cells when conducted by the same protocol in different laboratories, with the results eventually compiled in the same study or reproduced in follow-up studies, e.g., for TPA (No. 281) [78,186,259], 1-(No. 140) and 2-methylanthracene (No. 146) [78,89,194,195], fluoranthene (No. 124) [186,194,196,199,200,259], PCB 153 (No. 208) [78,90,200], microcystin-LR (No. 262) [314], methoxychlor (No. 88) or vinclozolin (No. 94) [235] and others. 

### 5.3. Comparison of SL-DT Results with Data on Genotoxicity and Carcinogenicity

#### 5.3.1. Genotoxicity Data

Next, we compared the search results for the SL-DT assay in WB-F344 cells with other assays and available data evaluating genotoxicity or carcinogenicity. Regarding genotoxicity, we found that only 27 compounds assessed using the SL-DT assay in WB-F344 cells are also included in databases of negative or positive chemicals in the Ames bacterial assay [315,316]. Interestingly, all these compounds, except 5, inhibited GJIC in WB-F344 cells, while 15 of them were negative in the Ames bacterial assay and 1 (CdCl_2_, No. 71) produced an equivocal result. This finding might be attributed to the very different mechanisms targeted by the SL-DT and Ames assays, and some important limitations of the Ames test based on bacterial cells to predict mutagenesis in humans [317]. Except for DEHP (No. 283) and chlorobenzilate (No. 83), Ames-negative chemicals showed positive or equivocal results in other in vitro genotoxic assays that use cultured eukaryotic cells or in in vivo genotoxic assays [315,316]. The 12–13 compounds negative or equivocal in the Ames test or other genotoxicity assays, but inhibiting GJIC, included several compounds classified by International Agency for Research on Cancer (IARC) into Groups 1-2A carcinogens, such as CdCl_2_ (No. 71), 17β-estradiol (No. 323), dieldrin (No. 86) and malathion (No. 91), and IARC Group 2B carcinogens (DEHP, No. 283, ochratoxin A, No. 89, 2,4-dichlorophenoxyacetic acid, No. 80), as well as chemicals categorized as carcinogens by Comptox/ToxRefDB (methoxychlor, No. 88; chlorobenzilate, No. 83; pyrene, No. 132). It clearly indicates that the carcinogenicity of non-mutagenic and non-genotoxic chemicals needs to be further studied and addressed in carcinogenicity testing to evaluate their non-genotoxic effects.

#### 5.3.2. IARC Carcinogenicity

Carcinogenicity data provided by the IARC [318] exist for 72 chemicals assessed using the SL-DT assay in WB-F344 (Supplementary Table S1 and File S1). The relationship between the results of the SL-DT assay and available data on carcinogenicity was statistically analyzed (Table 3). Sensitivity (True Positive rate), specificity (True Negative rate) and accuracy are widely used statistics to describe in vitro test methods according to the OECD Guidance Document 211. The overall sensitivity of the SL-DT assay as a predictor of all IARC carcinogens (Group 1, 2A or 2B) is 77%, the specificity 45% and the accuracy is 64%. Its sensitivity to predict carcinogenic chemicals in humans (Group 1) remains similar (75%). Five IARC Group 1 carcinogens were false negatives in the WB-F344 cell-based SL-DT assay, specifically formaldehyde (No. 1) and PCB 77, 81, 126 and 169 (No. 185, 187, 201 and 214). These PCBs are the non-ortho-substituted and dioxin-like PCBs causing adverse effects through transcriptional responses mediated by the AhR [319]. Thus, as discussed in Section 5.1, they might need a longer time to exert their impact on in vitro models, but their GJIC-inhibitory activity (except PCB 126) was mostly evaluated after a short exposure (0.5–1 h) [90,207]. 

False negatives will probably decrease as we move forward with more comprehensive GJIC-carcinogenicity models. Many NGTxC have both direct and indirect effects on GJIC, such as PCBs and PAHs. For example, PCB 126 (No. 201) has minimal direct effects on GJIC [90] but is well known to induce the release of proinflammatory compounds, such as TNFα [320,321,322]. TNFα (No. 263) readily inhibits GJIC [59,199,323,324,325]. Thus, PCB 126 can dysregulate GJIC indirectly through TNFα and other proinflammatory cytokines. PAHs with bay or bay-like regions directly dysregulate GJIC [193,194,196] and induce the release of cytokines and eicosanoids that can inhibit GJIC through both paracrine- and autocrine-based mechanisms [76,89,326,327,328]. Thus, there is a need to assess carcinogenic potential via GJIC assays incorporating both direct (our present model) and indirect effects of compounds on GJIC and move into more comprehensive hazard assessment models. Further verification of indirect mechanisms of GJIC disruption would then require either more complex experimental designs, such as experiments with a conditioned medium from a population of ‘effector’ cells exposed to the chemical [329], or possibly more complex organotypic in vitro models for GJIC evaluation, such as co-cultures of multiple cell types or organoid models. In summary, complex mechanisms of disruption of tissue homeostatic control, which involve cell-specific effects and interactions of multiple cell types, will be needed and reflected in the eventual testing and computational approaches, especially for the correct interpretation of negative GJIC results.

Another example of a false negative is represented by a cyanobacterial toxin microcystin-LR (No. 262), which currently belongs to IARC Group 2B carcinogens (possibly carcinogenic to humans). Microcystin-LR also seems to contribute to tumor promotion via mechanisms not involving direct inhibition of GJIC in liver oval or progenitor cells such as WB-F344 [314]. Specifically, the effects of this highly potent hepatotoxin depend upon its cellular uptake, which is mediated by specific membrane transporters highly expressed in differentiated hepatocytes, but lowly expressed in less differentiated cells [330]. According to the available evidence, microcystin-LR at low concentrations (0.01–1 μM) elicits cytotoxicity, oxidative and genotoxic stress, and inflammatory responses primarily in terminally differentiated hepatocytes [330]. In contrast, comparable concentrations have only limited direct effects on other cell types [331,332]. Nevertheless, microcystin-induced hepatocyte damage, associated with releases of inflammatory and other signaling molecules and overall disruption of liver tissue homeostatic balance [331,332], can affect other cells and eventually lead to proliferative and tissue repair responses, including changes in GJIC.

The specificity of the SL-DT assay for the IARC carcinogens was relatively low. There are 16 false positives, including organochlorines methoxychlor (No. 88) and chlorobenzilate (No. 83), PAHs pyrene (No. 132), phenanthrene (No. 130) or fluoranthene (No. 124) and hydrogen peroxide (No. 265). Although these chemicals are currently not considered as carcinogenic by IARC, there are carcinogenicity warning data for most of them available in the CompTox/ToxRefDB database (Supplementary Table S1). Furthermore, tumor-promoting activity, NGTxC activity and epigenetic toxicity of many of these compounds, such as low molecular weight PAHs, are being discussed [326,327,333,334,335]. 

#### 5.3.3. ComTox/ToxRefDB Data

The ComTox/ToxRefDB database [336] gathers available carcinogenicity data from different sources. If there are no available data, not available is stated. That means we could calculate just the sensitivity for this database. Out of 82 chemicals tested in the SL-DT assay and indicated with carcinogenicity warning, 59 compounds inhibited GJIC in WB-F344 cells. The sensitivity of the SL-DT assay to predict the ComTox carcinogenicity data is, therefore, similar to the IARC carcinogens, i.e., 73% (Table 3). A total of 23 chemicals listed with the carcinogenic warning in the ComTox/ToxRefDB, but recognized as false negatives by the SL-DT assays, included again those five IARC Group 1 chemicals, i.e., formaldehyde (No. 1) and PCB 77, 81, 126 and 169 (Nos. 185, 187, 201, 214), and IARC Group 2B compounds indeno [1,2,3-cd]pyrene (No. 126), dibenzo[a,i]pyrene (No. 121), benzo[j]fluoranthene (No. 110), benzo[k]fluoranthene (No. 111) and microcystin-LR (No. 262).

#### 5.3.4. OncoLogic System

Another data source for the carcinogenicity of chemicals we used to compare results of the SL-DT assay was the US EPA predictive program OncoLogic [337]. OncoLogic is an expert system for predicting the potential carcinogenicity of chemicals, which combines structure–activity relationship (SAR) analysis and expert judgment by incorporating knowledge about mechanisms of action, metabolism and human epidemiological studies. OncoLogic calculated the predicted carcinogenic potential (a level of concern) for 143 compounds which were also tested by the SL-DT assay in WB-F344 cells. Compounds evaluated with higher than marginal or low carcinogenicity were considered as positive results. Compounds with low, marginal or equivocal results were considered as negatives. As summarized in Table 3, the specificity of the SL-DT assay is relatively good (67%), but the accuracy and sensitivity are quite low (50% and 23%, respectively). Interestingly, false positives, i.e., compounds positive in GJIC assay but negative in OncoLogic, also included chemicals viewed as carcinogenic by both IARC (1-2B) or ComTox/ToxRefDB, such as MBOCA (No. 40), 2,4-dichlorophenoxyacetic acid (No. 80), dieldrin (No. 86), ochratoxin A (No. 89), benzo[b]fluoranthene (No. 104), 7H-dibenzo[c,g]carbazole (No. 164) or just the CompTox/ToxRefDB (dicofol, No. 85, benzo[ghi]perylene, No. 109, fluorene, No. 125, phenanthrene, No. 130, pyrene, No. 132).

#### 5.3.5. Other Assays for In Vitro GJIC Assessment

The metabolic cooperation assay using Chinese hamster V79 cells is the only GJIC method whose predictivity for tumor promotors and carcinogens has been evaluated and published [338,339]. The sensitivity, specificity and accuracy of the metabolic cooperation assay for carcinogenic data from IARC or NTP (National Toxicology Program) in 2002 were 49%, 63, and 54%, respectively. Just 31 chemicals from our included studies were among 468 chemicals evaluated in metabolic cooperation assay [338,339]. The sensitivity of the SL-DT assay to predict the results from the metabolic cooperation assay is 100% (Table 3). However, the specificity is quite low (31%). Interestingly, 9 out of 11 chemicals (DEHP, No. 283; CaCl_2_, No. 71; TCDD, No. 259; benzo[a]pyrene, No. 102; 7,12-dimethylbenz[a]anthracene, No. 98; benz[a]anthracene, No. 100; ochratoxin A, No. 89; 17β-estradiol, No. 323; hydrogen peroxide, No. 265) that were positives in the SL-DT assay but negatives in the metabolic cooperation assay (i.e., false positives in this comparison), are the IARC carcinogens and/or with carcinogenicity supporting data available in the CompTox/ToxRefDB. For the two remaining chemicals (EGF, No. 261; gossypol, No. 305), carcinogenicity data are not available.

## 6. Conclusions and Future Perspective

The data analysis of our systematic search revealed that sensitivity (True Positive rate) of the SL-DT assay in WB-F344 cells for carcinogenicity, as provided by reputable carcinogen classifications and tools (e.g., IARC, ComTox/ToxRefDB, OncoLogic), is relatively good (67–77%). There seem to be plausible mechanistic explanations for several notable false negatives, which could be addressed by utilizing more comprehensive testing approaches and the assay within a testing strategy. The specificity (True Negative rate) of the assay is relatively low (45% for IARC carcinogens, 23% for OncoLogic). However, the lack of specificity appears to be an overarching issue in carcinogenicity assessment by individual tests, including in vivo and in vitro methods [3,15]. This can be addressed by improved mechanistic understanding, integration into mechanism-based testing approaches and strategies combining methods covering multiple traits and pivotal events, which would allow to better translate the results from in vitro tests and increase their predictivity towards humans [7].

The use of the SL-DT assay, and specifically its recent modification dubbed mSL-DT [259], and in combination with WB-F344 cell line, includes the following strengths: (1) it is relatively easy, simple and does not require special/expensive equipment or skills, (2) it has a low cost of supplies, and the dye solution can be re-used, and (3) it allows for the assessment of GJIC in a large population of the cells. (4) The assay has been successfully adapted for a microplate format, which allows for various degrees of automation, including cell and liquid handling steps, automated imaging and image analysis to improve the assay throughput. (5) The SL-DT assay can be combined with additional fluorophores, allowing for better quality control of the assay, evaluation of additional endpoints and more complex interpretation of the observed effects on GJIC. (6) The assay is also adaptable for various cell lines/types, as long as they are GJIC-competent and capable of growing in confluent monolayers. (7) In the case of WB-F344 cells, it uses a normal, noncancerous/nontumorigenic diploid cell line, which (8) has the potential to be differentiated in vitro to hepatocyte-like cells. Nevertheless, the SL-DT assay is also suitable for other cultures of adherent cells and cell lines. The assay is also suitable for (9) potential in vivo/ex vivo validation of the results by incision loading-dye transfer assay.

In contrast, this assay has some limitations. (1) This cell line primarily reflects tumor-promoting mechanisms involving Cx43-expressing liver epithelial/progenitor cells, as with most studies that have explored Cx43 in this cell line (Table 1). WB-F344 cells also express Cx26 (another Cx found in the healthy liver [340]), but to a lesser extent than Cx43 [309,341,342]. In contrast, the expression of Cx32, the major Cx type in hepatocytes, was not detected in these cells [309,343]. Thus, the lack of metabolic competence might not detect/predict tumor-promoting mechanisms affecting other cell types, for example, Cx32/Cx26-expressing hepatocytes that have retained the ability to divide, while the more mature hepatocytes play a greater role in metabolic functions in the liver and are not target cells for tumor neogenesis. Therefore, assessment of the chemical effects on GJIC mediated through other Cx types or Cx43 but within a different context (e.g., different cell types or tissue) would require the use of another relevant cell line. (2) WB-F344 represents a rodent cell line. The effects of several well-recognized GJIC inhibitors (e.g., TPA, 1-methylanthracene) observed in WB-F344 cells or other rodent cell lines were also reproduced in some studies with human cells and cell lines, e.g., [66,76,261,269,326,327,328,329,344,345]. However, the number of such studies remains relatively low, and there is a need for further research focusing on GJIC assessment using human cells. Due to the applicability of the (m)SL-DT assay towards different cell lines and cell types, the results obtained from WB-F344 cells could be relatively easily compared and/or validated in other in vitro models using the same methodology. (3) In the current setup, the assay is not well equipped to address eventual indirect mechanisms of GJIC inhibition, e.g., due to paracrine signaling through pro-inflammatory cytokines. Such mechanisms would need to be addressed by the suitable testing strategies combining multiple assays to evaluate other relevant mechanisms and endpoints and/or using more advanced complex in vitro models. (4) This SL-DT setup is not suitable to study GJIC in the individual cell pairs or the cells growing at low cell densities/low confluency and 3D cultures, where alternative techniques for GJIC assessment need to be applied. (5) Its use for assessing GJIC in the cells with irregular shaped/morphology (e.g., neurons) is rather complicated. Finally, (6) this method currently relies on the manual cutting step, which could eventually be automated/robotized.

Overall, the SL-DT assay represents a well-established technique repeatedly used in scientific toxicological and biomedical research by numerous laboratories across the world, where it has also been successfully applied to characterize alterations of GJIC by several hundreds of chemicals, including NGTxC and tumor promoters. The assay has a great potential to be further developed, improved, validated and subsequently utilized as a screening/testing tool within integrated testing approaches for NGTxC.

## 7. Literature Search and Statistical Measures

We systematically searched PubMed, SCOPUS and Web of Science for relevant published studies until 30 October 2020. The literature strategy for each database is in Supplementary Text S1A–C—the search strategy retrieved 2257 records without duplicates (Supplementary Figure S1). First, we screened the title and abstract of these 2257 studies using a web and mobile application Rayyan for systematic reviews [346] and excluded 2034 studies based on excluding criteria. Then, we assessed 223 full-text articles for their eligibility and 86 papers were included for further analysis. Finally, the quality of the included in vitro studies was evaluated using the SciRap in vitro web-based tool (version 1.0) [313].

We extracted the chemicals assessed for GJIC using SL-DT in the WB-F344 cell line and their GJIC inhibitory potential (positive, negative, equivocal) with the EC_50_ and ET_50_ values from the included papers (Supplementary Figure S1). We also added Chemical Abstracts Service Registry Number (CASRN) as a unique numerical identifier assigned by the Chemical Abstracts Service (CAS). Additionally, we include the GJIC-inhibitory potential of the extracted chemicals assessed using metabolic cooperation assay in V79 cells [338,339], their genotoxic activity obtained from the EURL ECVAM databases of Ames positive and negative compounds [315,316] and carcinogenicity potential reported by the IARC [318], proposed by the CompTox Chemistry Dashboard/ToxRefDB database [336,347] or predicted using US EPA OncoLogic™ 9/8 [337].

The IARC classifies compounds (1029 agents so far) as Group 1 (carcinogenic to humans: 121 agents), Group 2A (probably carcinogenic to humans: 89 agents), Group 2B (possibly carcinogenic to humans: 319 agents) and Group 3 (not classifiable as to its carcinogenicity to humans: 500 agents) and published data in the IARC Monographs, Volumes 1–129.

The CompTox/ToxRefDB database reports the cancer information of chemicals, including the availability of calculated cancer slope factor or inhalation unit risk and carcinogenicity data such as the IARC group, EPA OPP (Office of Pesticide Programs) cancer classes, NTP (National Toxicology Program) Reports on Carcinogens, NLM (National Library of Medicines) ToxNet (Toxicology Data Network) HSDB (Hazardous Substances Data Bank) or University of Maryland carcinogenicity warnings. If at least one piece of information was positive, we classified this chemical as positive (+). If there no supporting information is available, we classified it as data not available (NA).

OncoLogic™ uses the mechanism-based structure–activity relationships (SAR) analysis and incorporates expert judgment on available data. The structure-depending information is based on a variety of sources, including (a) “Chemical Induction of Cancer” Series (7 volumes, Academic Press, 1968–1995, by J.C. Arcos, M.F. Argus, Y.-t. Woo, and D.Y. Lai.), (b) IARC monograph series, (c) U.S. National Cancer Institute (NCI)/NTP technical report series, (d) PHS Publication No. 149: “Survey of Compounds Which Have Been Tested for Carcinogenic Activity” and (e) non-classified chemical industry and US EPA research data. The OncoLogic™ defines the six cancer concern levels in order from the lowest concern to the highest concern: (1) Low (unlikely to be a carcinogen), (2) Marginal (likely to have equivocal carcinogenic activity), (3) Low-moderate (likely to be weakly carcinogenic), (4) Moderate (likely to be a moderately active carcinogen), (5) Moderate-high (highly likely to be a moderately active carcinogen) and (6) High (highly likely to be a potent carcinogen). OncoLogic™ version 8.0 evaluates fibers, metals and polymers and OncoLogic™ version 9.0 more than 52 classes of organic chemicals.

Sensitivity (true positive rate) was calculated as true positives divided by the sum of true positives and false negatives. Specificity (true negative rates) was calculated as true negatives divided by the sum of true negatives and false positives. Finally, accuracy was calculated as the proportion of true results, either true positive or true negative, in all assessed chemicals.

## Figures and Tables

**Table 2:** Setups compatible for HTS and/or HCA/HCS of gap junctional intercellular communication (GJIC) (adapted from [259]).

Method	Tracer	Cell Line	Set-Up	HTS/HCA/HCS Features	Advantages +/Disadvantages −
Dye transfer assays					
Multiparametric SL-DT assay [259]	LY	WB-F344 HBE1 Beas-2B TM3 TM4	24-96-wp	Platform ready for automated cell seeding or compound adding. Automatic fixed cell imaging.	+ Endpoints: GJIC, cell density and viability + Applicable for a variety of adherent cell types + Automated image acquisition and analysis + No specialized cell model, equipment or technical skills needed − Invasive − For cells forming nearly confluent monolayers − Not applicable for Cx channels excluding LY
Microinjection [280]	HPTS	HeLa HEK293 HL-1	35-mm Petri dish	Robotic microinjection system. Automatic live cell imaging.	+ Precise and quantitative + Instantaneous delivery + Automated microinjection of a large of cells + Permits the correlation of morphological + Enables kinetic studies (the transfer rate from one cell to another) and functional data from individual cells − Manual image analysis − Low throughput/time consuming − Specialized microinjection equipment needed − Invasive − Unsuitable for rapid effects or requiring a continuous application of the stimulus
Electroporation [281]	LY	Cx43-C6	Electroporation slides	Manual live-cell imaging by light and fluorescence microscopy. Low throughput.	+ A rapid and objective quantification with a high degree of reproducibility + Applicable to a large variety of adherent cell types + Semiautomatic image analysis − A specialized electroporation equipment needed − Low throughput − Unsuitable for poorly adherent cells − Cell health concerns − Not applicable for Cx channels excluding LY
GNOME-LP/DT [282]	LY	GM-7373 RBE4	24-96-wp	Automatic fixed cell imaging.	+ Automated image analysis + Non-invasive + Semiautomatic image analysis + Applicable for 3D models or tissue − Low throughput − Inappropriate to investigate cell assemblies or low-density cultures − Specialized microscopic equipment needed − Not applicable for Cx channels excluding LY
Parachute assay	Calcein [243]	WB F344	96-wp	Platform ready for automated cell seeding or compound adding. Automatic live cell imaging.	+ Automated image acquisition and analysis + Semiautomated HCS − Relatively long (2–5 h) time-lapse image acquisition − Nonspecific dye transfer of calcein has been observed [97] − Calcein can be actively pumped out by MRPs [283] − Trypsinization of the donor cells − Formation of GJ channels between donor and recipient cells during exposure
	Calcein/Vybrant DiI [284]	U251			
Preloading assay	Calcein	IAR-20 [139]	384-wp	Automated cell seeding. Automated compound adding.	+ Automated image acquisition and analysis + The high level of automation with very few manual steps − Special live microscopy equipment needed − Relatively long (2 h) time-lapse image acquisition − Nonspecific dye transfer of calcein has been observed [97] − Calcein can be actively pumped out by MRPs [283] − Trypsinizations of the preloaded cells − Formation of GJ channels between donor and recipient cells during (or after) exposure
		Cxs-C6 [285]			
Microfluidic assay	CFDA [286]	NRK-49F	Chip	Microfluidic platform. Automatic live cell imaging.	+ Non-invasive + In situ monitoring of GJIC + Rapid screening + Applicable for studying the kinetics of gap junction channels diffusion + Ease-of-use + Can be scaled up for high-throughput applications + Low reagent consumption − Specialized microfluidic equipment needed − Only suitable for monolayer forming cells − A limited number of studied cells − Nonspecific dye transfer of calcein has been observed [97] − Calcein can be actively pumped out by MRPs [283]
	Calcein [287]	C6 Cx43-C6 HeLa			
Metabolic cooperation assays					
Cx43 GJ aequorin assay [288]	Calcium	HeLa CHO HEK29 U2OS (transduced with Cx43 and cytoAeq/α1-ARs or TRPV1)	384-wp	Platform ready for automated cell seeding and compound adding. Luminescence plate reader.	+ HTS screening assay (miniaturization) + Non-invasive + Does not require complex equipment and analysis − Genetically modified cells needed − High probability of false-positive hits − Optimized only for Cx43
I--YFP^QL^ assay [289,290]	Iodide	LN215 HOS (transduced or non-transducted with iodide transporter/iodide sensor protein)	96-wp	Platform ready for automated cell seeding and compound adding. Fluorescence plate reader.	+ HTS screening assay (miniaturization) + Non-invasive + Short assay time (10 s) + Does not require complex equipment and analysis − Genetically modified cells needed − High probability of false-positive hits − Optimized only for Cx43
cAMP based assay [291]	cAMP	HeLa (transfected with Cx43 and A2AAR or GloSensor-20F)	96-wp	Platform ready for automated cell seeding and compound adding. Luminescence plate reader.	+ HTS screening assay (miniaturization) + Non-invasive + Short assay time (1 s) + Does not require complex equipment and analysis + Applicable for studying the kinetics of gap junction channels diffusion − Genetically modified cells needed − High probability of false-positive hits − Optimized only for Cx43

Abbreviations: **α1-ARs**, α-1 adrenergic receptor; **A2AAR**, Gs protein-coupled adenosine A_2A_ receptor; **Beas-2B**, human bronchial epithelial cell line (ATCC CRL-9609); **C6**, rat malignant glioma cell line (ATCC CCL-107™); **CFDA**, carboxyfluorescein diacetate; **CHO**, Chinese hamster ovary cells (RRID:CVCL_0213); **Cx**, connexin; **Cx43-C6**, C6 cells stably transfected with Cx43; **Cxs C6**, C6 cells stably expressing channels of different connexins; **cytoAeq**, calcium-sensitive luminescent protein aequorin enhanced by codon optimization; **GJC**, gup junction channel; **GloSensor-20F**, cAMP-sensing GloSensor luciferase; **GM-7373**, tumorigenic bovine aortic endothelial cell line (DSMZ ACC 109); **GNOME-LP/DT**, nanoparticle-mediated laser perforation-dye transfer; **HBE1**, immortalized human bronchial epithelial cell line (RRID:CVCL_0287; Kerafast #ENC002); **HCA**/**HCS**, high-content analysis/screening; **HEK293**, human embryonic kidney 293 cells (ATCC CRL-1573); **HeLa**, human cervical cancer cell line (RRID:CVCL_0030); **HOS**, human bone osteosarcoma cells (RRID:CVCL_0312); **HL-1**, mouse cardiac muscle cells (RRID:CVCL_0303); **HPTS**, 8-hydroxypyrene-1,3,6-trisulfonic acid; **HTS**, high-throughput screening; **IAR-20**, non-transformed rat liver epithelial cells (RRID:CVCL_5296); **LN215**, human astroglioma cells (RRID:CVCL_3954); **MRPs**, multidrug resistance proteins; **NRK-49F**, normal rat kidney fibroblasts (ATCC CRL-1570); **RBE4**, immortalized rat brain endothelial cells (RRID:CVCL_0495); **TM3**, murine immortalized immature Leydig cell line (ATCC CRL-1714^TM^); **TM4**, murine immortalized immature Sertoli cell line (ATCC CRL-1715^TM^); **U251**, human glioblastoma-derived cell line (RRID:CVCL_0021); **U2OS**, human bone osteosarcoma epithelial cells (ATCC HTB-96); **WB-F344**, normal rat liver epithelial cell line (RRID:CVCL_9806; JCRB0193); **wp**, well plate.

**Table 3:** Comparison between carcinogenicity evaluated by the IARC, CompTox, OncoLogic or the metabolic cooperation (MC) and the SL-DT assay in WB-F344 cells. In the table, number of assessed chemicals are given, and the SL-DT assay sensitivity and (if applicable) specificity and accuracy (%) are provided. Raw data are provided in the Supporting Information.

		SL-DT Assay		Total Chemicals
Carcinogenicity		↓ ^a^	— or —/↓ or E ^b^	
IARC	Group 1, 2A and 2B	33	10	72
	Group 3	16	13	
	Sensitivity	77% (33/43)		
	Specificity	45% (13/29)		
	Accuracy	64% (46/72)		
	Group 1	15	5	20
	Sensitivity	75% (15/20)		
CompTox	+ ^c^	59	23	82
	Sensitivity	73% (60/82)		
OncoLogic	Low-moderate, Moderate, Moderate-high, High	58	29	143
	Low, Marginal, Marginal to High-moderate, Low to Moderate to Marginal, Low to Moderate, Marginal to Low moderate	43	13	
	Sensitivity	67% (58/87)		
	Specificity	23% (13/56)		
	Accuracy	50% (71/143)		
MC Assay	↓ ^a^	15	0	31
	—, E ^b^	11	5	
	Sensitivity	100% (15/15)		
	Specificity	31% (5/16)		
	Accuracy	65% (20/31)		

^a^ [↓]: GJIC inhibiting chemicals; ^b^ [—]: chemicals not inhibiting GJIC, or [—/↓] chemicals with majority of studies showing no GJIC inhibiting activity, or [E] chemicals with equivocal data on GJIC inhibition; ^c^ [+]: Categorized as carcinogens by CompTox. Red or Green background indicate either “Positive” or “Negative” classification for calculation of Sensitivity (True Positive Rate), Specificity (True Negative Rate) and Accuracy.

## Data Availability

Data supporting the reported results are available upon reasonable request to the corresponding authors.

## Supplementary Materials

The following are available online at https://www.mdpi.com/article/10.3390/ijms22168977/s1, Text S1: Literature strategy, Figure S1: A study flow for the systematic literature search, Table S1: Results of 328 chemicals assessed using SL-DT assay in WB-F344 cells; File S1: The Excel file containing the database containing 328 chemicals evaluated using the SL-DT assay in WB-F344 cells; File S2: The Excel file containing the quality study evaluation using in vitro Sci Rap web-based tool.
