# Supplementary material for: Applicability of Scrape Loading-Dye Transfer Assay for Non-Genotoxic Carcinogen Testing
Source: Int J Mol Sci. 2021 Aug 20;22(16):8977. doi: 10.3390/ijms22168977 (PMC8396440; doi:10.3390/ijms22168977)
Supplement: Supplementary file 1 [file ijms-22-08977-s001.zip › Supplementary_Information.pdf]

International Journal of
Molecular Sciences

MDPI

Review

Applicability of Scrape Loading-Dye Transfer Assay for Non-Genotoxic Carcinogen Testing

Iva Sovadinová [1], Brad L. Upham [2], James E. Trosko [2] and Pavel Babica [1,*]

1 RECETOX, Faculty of Science, Masaryk University, 625-00 Brno, Czech Republic; iva.sovadinova@recetox.muni.cz
2 Department of Pediatrics and Human Development, Institute for Integrative Toxicology, Michigan State University, East Lansing, MI 48824, USA; upham@msu.edu (B.L.U.); trosko@msu.edu (J.E.T.)
* Correspondence: pavel.babica@recetox.muni.cz; Tel.: +420-549-494-620

Content

**Suplementary Texts**	Pages
Suplementary Text S1	S2-S4
**Suplementary Tables**	
Suplementary Table S1	S5-S25
**Suplementary Figures**	
Suplementary Figure S1	S26
**References**	S27-S33

Supplementary Texts

Supplementary Text S1

A. Literature strategy in PubMed through October 30, 2020

(("Chemicals and Drugs Category"[MeSH Terms] OR "Xenobiotics"[MeSH Terms]) OR "Inorganic Chemicals"[MeSH Terms]) OR "Organic Chemicals"[MeSH Terms]) OR "Pharmaceutical Preparations"[MeSH Terms]) OR "Polycyclic Compounds"[MeSH Terms]) OR "Heterocyclic Compounds"[MeSH Terms]) OR "Pesticides"[MeSH Terms]) OR "toxins, biological"[MeSH Terms]) OR "radiation, ionizing"[MeSH Terms]) OR "agent*"[Text Word]) OR "anthracene*"[Text Word]) OR "aromatic polycyclic hydrocarbon*"[Text Word]) OR "bisphenol*"[Text Word]) OR "BPA"[Text Word]) OR "BPF"[Text Word]) OR "BPS"[Text Word]) OR "brominated flame retardant*"[Text Word]) OR "Cadmium"[Text Word]) OR "Carbon"[Text Word]) OR "Carbon Tetrachloride"[Text Word]) OR "carcinogen*"[Text Word]) OR "chemical*"[Text Word]) OR "Chlorpyriphos"[Text Word]) OR "compound*"[Text Word]) OR "contaminant*"[Text Word]) OR "DBP"[Text Word]) OR "DDE"[Text Word]) OR "DDT"[Text Word]) OR "DEHP"[Text Word]) OR "Dibutyl phthalate"[Text Word]) OR "Dieldrin"[Text Word]) OR "Diethylhexyl"[Text Word]) OR "Diethylhexyl phthalate"[Text Word]) OR "DINCH"[Text Word]) OR "dioxin*"[Text Word]) OR "drug*"[Text Word]) OR "fluorene*"[Text Word]) OR "Furan"[Text Word]) OR "metal*"[Text Word]) OR "naphthalene*"[Text Word]) OR "organophosphate*"[Text Word]) OR "Paraben"[Text Word]) OR "PCB"[Text Word]) OR "PCBs"[Text Word]) OR "Perchlorate"[Text Word]) OR "perfluorinated chemical*"[Text Word]) OR "pesticide*"[Text Word]) OR "PFOA"[Text Word]) OR "PFOS"[Text Word]) OR "Phenol"[Text Word]) OR "phthalate*"[Text Word]) OR "phytoestrogen*"[Text Word]) OR "pollutant*"[Text Word]) OR "polychlorinated biphenyl*"[Text Word]) OR "polychlorinated dibenzodioxin*"[Text Word]) OR "polycyclic aromatic hydrocarbon*"[Text Word]) OR "polynuclear aromatic hydrocarbon*"[Text Word]) OR "pyrene*"[Text Word]) OR "TCDD"[Text Word]) OR "Tetrachloride"[Text Word]) OR "Tetrachloromethane"[Text Word]) OR "Tetracycline"[Text Word]) OR "Thioacetamide"[Text Word]) OR "Toxicant"[Text Word]) OR "toxin*"[Text Word]) OR "Tributyltin"[Text Word]) OR "Triclosan"[Text Word]) OR "tumor promotor*"[Text Word]) OR "volatile organic compound*"[Text Word]) OR "xenobiotic*"[Text Word]) OR "Heterocyclic Compounds"[Text Word]) OR "Inorganic Chemicals"[Text Word]) OR "Organic Chemicals"[Text Word]) OR "Pesticides"[Text Word]) OR "Pharmaceutical Preparations"[Text Word]) OR "Polycyclic Compounds"[Text Word]) OR "Biological Toxins"[Text Word]) OR "Xenobiotics"[Text Word]) OR "Ionizing Radiation"[Text Word]) OR "Ionizing Radiations"[Text Word]) AND ((((((((((("Gap Junctions"[MeSH Terms] OR "Cell Junction"[Text Word]) OR "Cell Junctions"[Text Word]) OR "Gap Junction Channel Proteins"[Text Word]) OR "Gap Junction Protein"[Text Word]) OR "Gap Junction Proteins"[Text Word]) OR "Gap Junction"[Text Word]) OR "Gap junctional intercellular communication"[Text Word]) OR "Gap junctional intercellular communications"[Text Word]) OR "Gap Junctions"[Text Word]) OR "Gap Junctions"[Text Word]) OR "GJIC"[Text Word])) AND ((("Hepatocytes"[MeSH Terms] OR "primary liver cell*"[Text Word]) OR "liver"[Text Word]) OR "hepatic"[Text Word]) OR "oval cell*"[Text Word]) OR "hepatic cell*"[Text Word]) OR "sinusoidal endothelial cell*"[Text Word]) OR "kupffer cell*"[Text Word]) OR "stellate cell*"[Text Word]) OR "liver endothelial cell*"[Text Word]) OR "liver epithelial cell line"[Text Word]) OR "liver slice"[Text Word]) OR "parenchymal cell*"[Text Word]) OR "nonparenchymal cell*"[Text Word]) OR "non parenchymal cell*"[Text Word]) OR "liver cell*"[Text Word]) OR "hepatocyte*"[Text Word]) OR "ito cell*"[Text Word]) OR "ARL 18"[Text Word]) OR "ARL18"[Text Word]) OR "Ac2F"[Text Word]) OR "AH601"[Text Word]) OR "AH66tc"[Text Word]) OR "AH70Btc"[Text Word]) OR "alexander cell*"[Text Word]) OR "ARLJ301-3"[Text Word]) OR "AML12"[Text Word]) OR "fah cell*"[Text Word]) OR "Hep3B"[Text Word]) OR "F344"[Text Word]) OR "wb f344"[Text Word]) OR "wb f344"[Text Word]) OR "WBF344"[Text Word]) OR "wb cell*"[Text Word]) OR "WB-a/32-10"[Text Word]) OR "WB-aB1"[Text Word]) OR "WBHa-ras"[Text Word]) OR "Chang liver"[Text Word]) OR "MTR6"[Text Word]) OR "T51B"[Text Word]) OR "BRL"[Text Word]) OR "BRL-3A"[Text Word]) OR "mRLh"[Text Word]) OR "dRLa-74"[Text Word]) OR "dRLh"[Text Word]) OR "dRLN"[Text Word]) OR "FLS"[Text Word]) OR "Hep G2"[Text Word]) OR "HepG2"[Text Word]) OR "Huh7"[Text Word]) OR "Huh"[Text Word]) OR "Huh-7"[Text Word]) OR "HLE"[Text Word]) OR "HLF"[Text Word]) OR "HuCCT1"[Text Word]) OR "JHH"[Text Word]) OR "JTC-16"[Text Word])

OR "P3"[Text Word]) OR "LMH"[Text Word]) OR "M.P3"[Text Word]) OR "NCTC clone"[Text Word]) OR "NOZ"[Text Word]) OR "OCUG-1"[Text Word]) OR "PLC/PRF/5"[Text Word]) OR "RI-T"[Text Word]) OR "RL-34"[Text Word]) OR "RLC"[Text Word]) OR "RLN"[Text Word]) OR "TLR"[Text Word]) OR "T51B"[Text Word]) OR "rel cell*"[Text Word]) OR "IAR20"[Text Word]) OR "IAR 20"[Text Word]) OR "IAR203"[Text Word]) OR "IAR27E"[Text Word]) OR "IAR6-1"[Text Word]) OR "IAR27F"[Text Word]) OR "IAR"[Text Word]) OR "Clone 9"[Text Word]) OR "RLEC"[Text Word]) OR "H4IIE"[Text Word]) OR "MH1C1"[Text Word]) OR "MIHA"[Text Word]) OR "HepaRG"[Text Word])

B. Literature strategy in SCOPUS through October 30, 2020

TOPIC: ("primary liver cell*" OR "liver" OR "hepatic" OR "oval cell*" OR "hepatic cell*" OR "sinusoidal endothelial cell*" OR "Kupffer cell*" OR "stellate cell*" OR "liver endothelial cell*" OR "liver epithelial cell line" OR "liver slice" OR "parenchymal cell*" OR "nonparenchymal cell*" OR "non-parenchymal cell*" OR "liver cell*" OR "hepatocyte*" OR "Ito cell*" OR "ARL 18" OR "ARL18" OR "Ac2F" OR "AH601" OR "AH66tc" OR "AH70Btc" OR "AH-7974.P3" OR "Alexander cell*" OR "ARLJ301-3" OR "AML12" OR "FAH cell*" OR "CFH cell*" OR "Hep3B" OR "F344" OR "WB-F344" OR "WB F344" OR "WBF344" OR "WB cell*" OR "WB-a/32-10" OR "WB-aB1" OR "WBHa-ras" OR "Chang liver" OR "MTR6" OR "T51B" OR "BRL" OR "BRL-3A" OR "mRLh" OR "dRLa-74" OR "dRLh" OR "dRLN" OR "FLS" OR "Hep G2" OR "HepG2" OR "Huh7" OR "Huh" OR "Huh-7" OR "HLE" OR "HLF" OR "HuCCT1" OR "JHH" OR "JTC-16" OR "P3" OR "LMH" OR "M.P3" OR "NCTC clone" OR "NOZ" OR "OCUG-1" OR "PLC/PRF/5" OR "RI-T" OR "RL-34" OR "RLC" OR "RLN" OR "TLR" OR "T51B" OR "REL cell*" OR "IAR20" OR "IAR 20" OR "IAR203" OR "IAR 203" OR "IAR27E" OR "IAR 27E" OR "IAR6-1" OR "IAR27F" OR "IAR" OR "Clone 9" OR "RLEC" OR "H4IIE" OR "MH1C1" OR "MIHA" OR "HepaRG") AND TOPIC: ("Chemicals and Drugs Category" OR "Xenobiotics" OR "Inorganic Chemicals" OR "Organic Chemicals" OR "Pharmaceutical Preparations"[Mesh] OR "Polycyclic Compounds" OR "Heterocyclic Compounds" OR "Pesticides" OR "Toxins" OR "Toxin" OR "Cyanotoxin" OR "Cyanotoxins" OR "Agent*" OR "Anthracene*" OR "Aromatic Polycyclic Hydrocarbon*" OR "Bisphenol*" OR "BPA" OR "BPF" OR "BPS" OR "Brominated Flame Retardant*" OR "Cadmium" OR "Carbon" OR "Carbon Tetrachloride" OR "Carcinogen*" OR "Chemical*" OR "Chlorpyriphos" OR "Compound*" OR "Contaminant*" OR "DBP" OR "DDE" OR "DDT" OR "DEHP" OR "Dibutyl phthalate" OR "Dieldrin" OR "Diethylhexyl" OR "Diethylhexyl phthalate" OR "DINCH" OR "Dioxin*" OR "Drug*" OR "Fluorene*" OR "Furan" OR "Metal*" OR "Naphthalene*" OR "Organophosphate*" OR "Paraben" OR "PCB" OR "PCBs" OR "Perchlorate" OR "Perfluorinated chemical*" OR "Pesticide*" OR "Herbicide*" OR "Insecticide*" OR "PFOA" OR "PFOS" OR "Phenol" OR "Phthalate*" OR "Phytoestrogen*" OR "Personal care product" OR "Personal care products" OR "Pollutant*" OR "Polychlorinated Biphenyl*" OR "Polychlorinated dibenzodioxin*" OR "Polycyclic Aromatic Hydrocarbon*" OR "Polynuclear Aromatic Hydrocarbon*" OR "Pyrene*" OR "TCDD" OR "Tetrachloride" OR "Tetrachloromethane" OR "Tetracycline" OR "Thioacetamide" OR "Toxicant*" OR "Tributyltin" OR "Triclosan" OR "Tumor promotor*" OR "Volatile Organic Compound*" OR "Xenobiotic*" OR "Heterocyclic Compounds" OR "Inorganic Chemicals" OR "Organic Chemicals" OR "Pesticides" OR "Pharmaceutical Preparations" OR "Polycyclic Compounds" OR "Biological Toxins " OR "Ionizing Radiation" OR "Ionizing Radiations") AND TOPIC: ("Cell Junction" OR "Cell Junctions" OR "Gap Junction Channel Proteins" OR "Gap Junction Protein" OR "Gap Junction Proteins" OR "Gap Junction" OR "Gap junctional intercellular communication" OR "Gap junctional intercellular communications" OR "Gap Junctions" OR "Gap Junctions" OR "GJIC")

Timespan: All years. Indexes: SCI-EXPANDED, SSCI, A&HCI, CPCI-S, CPCI-SSH, BKCI-S, BKCI-SSH, ESCI, CCR-EXPANDED, IC.

C. Literature strategy in Web of Science through October 30, 2020

TITLE-ABS-KEY ("primary liver cell*" OR "liver" OR "hepatic" OR "oval cell*" OR "hepatic cell*" OR "sinusoidal endothelial cell*" OR "Kupffer cell*" OR "stellate cell*" OR "liver endothelial cell*" OR "liver epithelial cell line" OR "liver slice" OR "parenchymal cell*" OR "nonparenchymal cell*" OR "non-parenchymal cell*" OR "liver cell*" OR "hepatocyte*" OR "Ito cell*" OR "ARL 18" OR "ARL18" OR "Ac2F" OR "AH601" OR "AH66tc" OR "AH70Btc" OR "AH-7974.P3" OR "Alexander cell*" OR "ARLJ301-3" OR "AML12" OR "FAH cell*" OR "CFH cell*" OR "Hep3B" OR "F344" OR "WB-F344" OR "WB F344" OR "WBF344" OR "WB cell*" OR "WB-a/32-10" OR "WB-aB1" OR "WBHa-ras" OR "Chang liver" OR "MTR6" OR "T51B" OR "BRL" OR "BRL-3A" OR "mRLh" OR "dRLa-74" OR "dRLh" OR

"dRLN" OR "FLS" OR "Hep G2" OR "HepG2" OR "Huh7" OR "Huh" OR "Huh-7" OR "HLE" OR "HLF" OR "HuCCT1" OR "JHH" OR "JTC-16" OR "P3" OR "LMH" OR "M.P3" OR "NCTC clone" OR "NOZ" OR "OCUG-1" OR "PLC/PRF/5" OR "RI-T" OR "RL-34" OR "RLC" OR "RLN" OR "TLR" OR "T51B" OR "REL cell*" OR "IAR20" OR "IAR 20" OR "IAR203" OR "IAR 203" OR "IAR27E" OR "IAR 27E" OR "IAR6-1" OR "IAR27F" OR "IAR" OR "Clone 9" OR "RLEC" OR "H4IIE" OR "MH1C1" OR "MIHA" OR "HepaRG") AND TITLE-ABS-KEY ("Chemicals and Drugs Category" OR "Xenobiotics" OR "Inorganic Chemicals" OR "Organic Chemicals" OR "Pharmaceutical Preparations" OR "Polycyclic Compounds" OR "Heterocyclic Compounds" OR "Pesticides" OR "Toxins" OR "Toxin" OR "Cyanotoxin" OR "Cyanotoxins" OR "Agent*" OR "Anthracene*" OR "Aromatic Polycyclic Hydrocarbon*" OR "Bisphenol*" OR "BPA" OR "BPF" OR "BPS" OR "Brominated Flame Retardant*" OR "Cadmium" OR "Carbon" OR "Carbon Tetrachloride" OR "Carcinogen*" OR "Chemical*" OR "Chlorpyriphos" OR "Compound*" OR "Contaminant*" OR "DBP" OR "DDE" OR "DDT" OR "DEHP" OR "Dibutyl phthalate" OR "Dieldrin" OR "Diethylhexyl" OR "Diethylhexyl phthalate" OR "DINCH" OR "Dioxin*" OR "Drug*" OR "Fluorene*" OR "Furan" OR "Metal*" OR "Naphthalene*" OR "Organophosphate*" OR "Paraben" OR "PCB" OR "PCBs" OR "Perchlorate" OR "Perfluorinated chemical*" OR "Pesticide*" OR "Herbicide*" OR "Insecticide*" OR "PFOA" OR "PFOS" OR "Phenol" OR "Phthalate*" OR "Phytoestrogen*" OR "Personal care product" OR "Personal care products" OR "Pollutant*" OR "Polychlorinated Biphenyl*" OR "Polychlorinated dibenzodioxin*" OR "Polycyclic Aromatic Hydrocarbon*" OR "Polynuclear Aromatic Hydrocarbon*" OR "Pyrene*" OR "TCDD" OR "Tetrachloride" OR "Tetrachloromethane" OR "Tetracycline" OR "Thioacetamide" OR "Toxicant*" OR "Tributyltin" OR "Triclosan" OR "Tumor promotor*" OR "Volatile Organic Compound*" OR "Xenobiotic*" OR "Heterocyclic Compounds" OR "Inorganic Chemicals" OR "Organic Chemicals" OR "Pesticides" OR "Pharmaceutical Preparations" OR "Polycyclic Compounds" OR "Biological Toxins " OR "Ionizing Radiation" OR "Ionizing Radiations") AND TITLE-ABS-KEY ("Cell Junction" OR "Cell Junctions" OR "Gap Junction Channel Proteins" OR "Gap Junction Protein" OR "Gap Junction Proteins" OR "Gap Junction" OR "Gap junctional intercellular communication" OR "Gap junctional intercellular communications" OR "Gap Junctions" OR "Gap Junctions" OR "GJIC")

Tables

Table S1 Results of 328 chemicals assessed using SL-DT assay in WB-F344 cells

No.	Chemical group / Chemical	CASRN	GJIC: SL-DT assay – ↓	EC50 (µM)	ET50 (min)	Ref.	MC assay [1,2]	Genotoxicity: Ames Overall [3,4]	Other assays [3,4]	Over all	Carcinogenicity: OncoLogic 9.0/8.0 [6]	IARC [7]	CompTox/ ToxRefDB [5,8]
	**Aldehydes**												
1	Formaldehyde	50-00-0	–	**>100** (0.5 h)	*n.d.*	[9]	–	+	+	+	**High-moderate**	**Group 1**	+
	**Anilides**												
2	Vinclozolin M2	83792-61-4	↓	**250** (2 h)	**120** (250 µM)	[10]							**N.A.**
	**Aromatic amine derivates**												
3	N-OH-MBOCA		–	**>4** (0.5 h)	*n.d.*	[11]							
4	NO-MBOCA		–	**>72** (0.5 h)	*n.d.*	[11]							
5	o-OH-MBOCA		–	**>180** (0.5 h)	*n.d.*	[11]							
	**Benzopyrans**												
6	Epigallocatechin gallate	989-51-5	↓	150 (1 h)	**150** (200 µM)	[12]							**N.A.**
	**Cannabinoids**												
7	Cannabinol	521-35-7	↓	**13** (0.5 h)	**10** (20 µM)	[13]							**N.A.**
8	THC	1972-08-3	↓	**13** (0.5 h)	**10** (20 µM)	[13]							+
	**Carbamates**												
9	Carbofuran	1563-66-2	–	**>452** (1 h)	*n.d.*	[14]					**Uncertain/ Unknown**		+
	**Catechins**												
10	Epicatechin	490-46-0	–	**>96** (1, 24 h)	*n.d.*	[12,15]							**N.A.**
	**Chalcone derivates**												

11	2',3',3-Trihydroxychalcone		↓	**181** (0.5 h)	*n.d.*	[26]					
12	2',3-Dihydroxychalcone		↓	**9** (0.5 h)	*n.d.*	[26]					
13	2',3-Dihydroxydihydrochalcone		↓	**74** (0.5 h)	*n.d.*	[26]					
14	2,4,4'-Trihydroxychalcone	83616-07-3	—	**>200** (0.5 h)	*n.d.*	[26]					**N.A.**
15	2',4',4-Trihydroxychalcone		—	**>200** (0.5 h)	*n.d.*	[26]					
16	2',4',4-Trihydroxydihydrochalcone		—	**>200** (0.5 h)	*n.d.*	[26]					
17	2',4'-Dihydroxy-3-methoxychalcone		—	**>200** (0.5 h)	*n.d.*	[26]					
18	2',4-Dihydroxy-3-methoxychalcone		↓	**140** (0.5 h)	*n.d.*	[26]					
19	2',4'-Dihydroxy-3-methoxydihydrochalcone		—	**>200** (0.5 h)	*n.d.*	[26]					
20	2',4-Dihydroxychalcone	13323-66-5	↓	**49** (0.5 h)	*n.d.*	[26]					**N.A.**
21	2',4-Dihydroxydihydrochalcone		—	**>200** (0.5 h)	*n.d.*	[26]					
22	2'-Hydroxy-2,4-dimethoxychalcone		↓	**33** (0.5 h)	*n.d.*	[26]					
23	2'-Hydroxy-2,4-dimethoxydihydrochalcone		↓	**40** (0.5 h)	*n.d.*	[26]					
24	2'-Hydroxy-3,4-dimethoxychalcone		↓	**76** (0.5 h)	*n.d.*	[26]					
25	2'-Hydroxy-3,4-dimethoxydihydrochalcone		—	**>200** (0.5 h)	*n.d.*	[26]					
26	2'-Hydroxy-3-methoxychalcone		↓	**34** (0.5 h)	*n.d.*	[26]					
27	2'-Hydroxy-3-methoxydihydrochalcone		↓	**60** (0.5 h)	*n.d.*	[26]					
28	2'-Hydroxy-4-methoxychalcone	3327-24-0	↓	**83** (0.5 h)	*n.d.*	[26]					
29	2'-Hydroxy-4-methoxydihydrochalcone		↓	**50** (0.5 h)	*n.d.*	[26]					
30	2'-Hydroxychalcone	888-12-0	↓	**8** (0.5 h)	*n.d.*	[26]					

31	2-Hydroxychalcone	644-78-0	↓	**9** (0.5 h)	*n.d.*	[26]					**Low-moderate to Moderate**		**N.A.**
32	2'-Hydroxydihydrochalcone		↓	**13** (0.5 h)	*n.d.*	[26]							
33	2-Hydroxydihydrochalcone		↓	**>200** (0.5 h)	*n.d.*	[26]							
34	4'-Hydroxy-2,4-dimethoxychalcone		↓	**190** (0.5 h)	*n.d.*	[26]							
35	4'-Hydroxy-2,4-dimethoxydihydrochalcone		↓	**>200** (0.5 h)	*n.d.*	[26]							
36	4'-Hydroxychalcone	2657-25-2	—	**>200** (0.5 h)	*n.d.*	[26]					**Low-moderate to Moderate**		**N.A.**
37	4-Hydroxychalcone	20426-12-4	—	**>200** (0.5 h)	*n.d.*	[26]					**Low-moderate to Moderate**		**N.A.**
38	4'-Hydroxydihydrochalcone		—	**>200** (0.5 h)	*n.d.*	[26]							
39	4-Hydroxydihydrochalcone		↓	**>200** (0.5 h)	*n.d.*	[26]							
	**Chloroanilines**												
40	MBOCA	101-14-4	↓	**12** (0.5 h)	*n.d.*	[11]		+	+	+	**Low to High-moderate**	**Group 1**	+
	**Chlorobenzilate metabolite**												
41	4,4′-Dichlorobenzohydrol		—	**>60** (*n.s.*)	*n.d.*	[23]							
	**Chlorohydroxyfuranones**												
42	3-Chloro-4-(dichloromethyl)-5-hydroxy-2(5H)-furanone	77439-76-0	↓	**8 or 18** (1, 2 h) **>18** (24 h)	**90** (18 µM)	[22,27]	↓	+	+	+	**Low-moderate to Moderate**	**Group 2B**	**N.A.**
43	3,4-Dichloro-5-hydroxy-2(5H)-furanone	766-40-5	↓	**40** (1, 12 h)	*n.d.*	[22]					**Marginal to Low-moderate**		**N.A.**
	**Coumarins**												

44	Dicumarol	66-76-2	↓	**<100** (1 h)	*n.d.*	[16]				**Low-moderate**	**+**
45	Warfarin	81-81-2	↓	**5000** (1 h)	*n.d.*	[16]	↓			**Moderate**	**N.A.**
	**Diarylmethanes**										
46	Diacylglycerol kinase inhibitor I	93076-89-2	↓	**<30** (0.2 h)	*n.d.*	[17]					**N.A.**
	**Ethylenediamines**										
47	BAPTA-AM	126150-97-8	↓	**3 to 20** (0.8 h)	*n.d.*	[18]					**N.A.**
	**Fatty acid derivates**										
48	Arachidonic acid, 15-Hydroperoxy		↓	**<20** (0.2 h)	*n.d.*	[19]					
49	Arachidonic acid, 15-Hydroxy		↓	**<20** (0.2 h)	*n.d.*	[19]					
50	Arachidonic acid, Methyl ester		—	**>20** (0.2 h)	*n.d.*	[19]					
	**Fatty acid esters**										
51	1-Monolaurin	142-18-7	↓	**<150** (0.2 h)	*n.d.*	[17]					**N.A.**
52	L-Ascorbic acid 6-palmitate	137-66-6	↓	**35** (0.5, 1 h)	*n.d.*	[20]					**N.A.**
	**Fatty acids**										
53	Arachidonic acid	506-32-1	↓	**5 or <70** (0.2, 0.3 h)	**2** (20 µM)	[17,19]	↓				**N.A.**
54	Decanoic acid	334-48-5	—	**>375** (0.3 h)	*n.d.*	[21]				**Marginal**	**N.A.**
55	Docosahexaenoic acid	6217-54-5	↓	**<20** (0.2 h)	*n.d.*	[19]					**N.A.**
56	Eicosanoic acid	506-30-9	—	**>20** (0.2 h)	*n.d.*	[19]					**N.A.**
57	Eicosapentaenoic acid	10417-94-4	↓	**<20** (0.2 h)	*n.d.*	[19]					**N.A.**
58	γ-Linolenic Acid	506-26-3	↓	**<20** (0.2 h)	*n.d.*	[19]	↓				**N.A.**
59	Octanoic acid	124-07-2	—	**>375** (0.3 h)	*n.d.*	[21]					**N.A.**
60	PUFA 23:4 (n-6)		↓	**>20** (0.2 h)	*n.d.*	[19]					

	**Furans**												
61	3-Chloro-4-methyl-5-hydroxy-2(5H)-furanone	112309-61-2	↓	**700** (1, 12 h)	*n.d.*	[22]					**Low to Moderate**		**N.A.**
62	3-Chloro-4-(chloromethyl)-5-hydroxyfuran-2(5H)-one	125974-08-5	↓	**25** (1, 12 h)	*n.d.*	[22]					**Low-moderate to Moderate**		**N.A.**
	**Ginsenosides**												
63	Ginsenoside-Rb2	11021-13-9	—	**>210** (1 h)	*n.d.*	[15]							**N.A.**
	**Halogenated Diphenyl Compounds**												
64	4,4′-Dichlorobenzophenone	90-98-2	—	**>30** (*n.s.*)	*n.d.*	[23]					**Low-moderate**		**N.A.**
	**Heterocyclic Compounds**												
65	Fenarimol	60168-88-9	—	**>50** (0.5 h)	*n.d.*	[23]	**E**				**Low-moderate**		**N.A.**
	**Hydroquinone derivates**												
66	Tetrachlorohydroquinone	87-87-6	—	**>75** (1 h)	*n.d.*	[24]					**Moderate**		**N.A.**
67	tert-Butylhydroquinone	1948-33-0	—	**>10** (0.5 h)	*n.d.*	[18]		—	**E**	**E**			**N.A.**
	**Hydroxybenzoic acids**												
68	Propyl gallate	121-79-9	—	**>200** (1 h)	*n.d.*	[25]							**+**
	**Inorganic compounds**												
69	Arsenic trioxide	15502-74-6	↓	**25** (8 h)	**1152** (13 µM)	[28]					**High**		**N.A.**
	**Lysophospholipids**												
70	Lysophosphatidic acid	325465-93-8	↓	**<25** (0.25 h)	*n.d.*	[29]							**N.A.**
	**Metals**												
71	Cadmium chloride	10108-64-2	↓	**25** (4 h)	**30** (200 µM)	[30]	—	**E**	**+**	**+**	**High**	**Group 1**	**+**
	**Monocarboxylic acid amides**												
72	Flutamide	13311-84-7	—	**>100** (0.5-24 h)	**>1440** (100 µM)	[10]					**Moderate**		**+**
73	DEET	134-62-3	↓	>1000 (0.5 h)	*n.d.*	[31]							**+**

	**Methoxychlor metabolites**												
74	HPTE	2971-36-0	↓	**100** (0.5-24 h)	**360** (100 µM)	[10]							**N.A.**
	**Nanoparticles**												
75	SWCNTs		—	**>26** (0.5, 24 h)	*n.d.*	[32]							**N.A.**
	**Organic peroxides**												
76	Benzoylperoxide	94-36-0	↓	**150** or **<200** (0.25, 0.3 h)	**10** (50 µM)	[17,33]						Group 3	+
77	Dicumyl peroxide	80-43-3	↓	**10** or **<50** (0.25, 0.3 h)	**10** (200 µM)	[17,33]							**N.A.**
78	Di-tert-butyl peroxide	110-05-4	—	**>200** (0.25 h)	*n.d.*	[33]							**N.A.**
	**Organobromine compounds**												
79	Bromopropylate	18181-80-1	↓	**<20** (0.5 h)	*n.d.*	[34]	↓						**N.A.**
	**Organochlorine compounds**												
80	2,4-Dichlorophenoxyacetic acid	94-75-7	↓	**>450** (1 h)	*n.d.*	[14]	↓	—	E	E	Low	Group 2B	+
81	Alachlor	15972-60-8	↓	**<186 to 149** (0.4, 1 h)	**10** (186 µM)	[14,17]							+
82	Atrazine	1912-24-9	—	**>463** (0-20 h)	**>20** (463 µM)	[14]		—	E	E	Low-moderate to Moderate	Group 3	+
83	Chlorobenzilate	510-15-6	↓	<25 (0.5 h)	*n.d.*	[23,34]	↓	—	I	—		Group 3	+
84	DDT (p,p' or o,p')	50-29-3 or 789-02-6	↓	**13 to 40** (0.25-1 h)	**35** (30 µM)	[17,18,31,34–36]	↓				Low-moderate to Moderate	Group 2A	+
85	Dicofol	115-32-2	↓	**<10 to 30** (0.5 h)	*n.d.*	[34]	↓				Low to Moderate	Group 3	+
86	Dieldrin	60-57-1	↓	**<25** or **<10** (0.5, 1 h)	*n.d.*	[11,37]	↓	—	E	E	Marginal to High-moderate	Group 2A	+
87	Lindane	58-89-9	↓	**43 to <70** (0.25-1 h)	**10** (52 µM)	[14,17,35]					Moderate	Group 1	+

88	Methoxychlor	72-43-5	↓	**10** (0.5 h)	**4** (25 µM)	[10]	↓	—	E	E		Group 3	+
89	Ochratoxin A	303-47-9	↓	**>4** (16 h)	**960** (3 µM)	[38]	—	—	+	E	Low	Group 2B	+
90	Pentachlorophenol	87-86-5	↓	**<50** (0.2, 0.3 h) **>40** (1-24 h)	*n.d.*	[17,24,35]					Moderate	Group 1	+
	**Organophosphates**												
91	Malathion	121-75-5	↓	**600** (0.25 h)	**35** (500 µM)	[31]		—	+	E		Group 2A	+
	**Organosulfonic acids**												
92	Octanesulfonic acid	3944-72-7	—	**>375** (0.3 h)	*n.d.*	[21]							N.A.
	**Organotin compounds**												
93	Triphenyltin chloride	639-58-7	↓	**3** (0.5 h)	**25** (4 µM)	[39]							+
	**Oxazolidinones**												
94	Vinclozolin	50471-44-8	↓	**126** (0.5 h)	**1** (250 µM)	[10]					Marginal		+
	**Oxoanions**												
95	Peroxynitrite	19059-14-4	↓	**<150** (0.07 h)	*n.d.*	[40]							N.A.
	**PAH metabolites**												
96	Benzo[a]pyrene-7,8-diol-9,10-epoxide	55097-80-8	↓	**0.1** (0.5 h)	**20** (1 µM)	[41]							
	**PAHs**												
97	4H-Cyclopentano[*def*] phenanthrene	203-64-5	↓	**23** (0.5 h)	*n.d.*	[47]					Low		N.A.
98	7,12-dimethylbenz[a]anthracene	57-97-6	↓	**21** (0.5 h)	*n.d.*	[47,48]	—	+	+	+	High-moderate		+
99	Anthracene	120-12-7	—/↓	**>350** (0.25-1.5 h)	*n.d.*	[42,43,45–47,49]					Marginal	Group 3	+
100	Benz[a]anthracene	56-55-3	↓	**>100** (0.5 h)	*n.d.*	[47]	—	+	E	E	Low-moderate	Group 2B	+
101	Benzo[a]perylene	191-85-5	—	**>100** (0.5 h)	*n.d.*	[47]					High-moderate		N.A.
102	Benzo[a]pyrene	50-32-8	↓	**10 to 100**	*n.d.*	[41,47–49]	—	+	+	+	High	Group 1	+

				(0.5-1.5 h)									
103	Benzo[b]chrysene	214-17-5	—	**>100** (1 h)	*n.d.*	[50]					**Marginal**	**Group 3**	**N.A.**
104	Benzo[b]fluoranthene	205-99-2	↓	**>100** (0.5 h)	*n.d.*	[47]					**Low**	**Group 2B**	**+**
105	Benzo[c]chrysene	194-69-4	↓	**111** (1 h)	*n.d.*	[50]					**Moderate**		**N.A.**
106	Benzo[c]phenanthrene	195-19-7	↓	**29** (0.5 h)	*n.d.*	[47]					**Low-moderate**	**Group 2B**	**N.A.**
107	Benzo[e]pyrene	192-97-2	**E**	**>100** or **>79** (0.5, 1.5 h)	*n.d.*	[47,49]	—				**Marginal**	**Group 3**	**+**
108	Benzo[g]chrysene	196-78-1	↓	**60** (1 h)	*n.d.*	[50]					**Moderate**	**Group 3**	**N.A.**
109	Benzo[ghi]perylene	191-24-2	↓	**>100** (0.5 h)	*n.d.*	[47]					**Marginal**	**Group 3**	**+**
110	Benzo[j]fluoranthen	205-82-3	—	**>100** (0.5 h)	*n.d.*	[47]						**Group 2B**	**+**
111	Benzo[k]fluoranthene	207-08-9	—	**>100** (0.5 h)	*n.d.*	[47]						**Group 2B**	**+**
112	Chrysene	218-01-9	↓	**>100** (0.5 h)	*n.d.*	[47]		+	+	+	**Low-moderate**	**Group 2B**	**+**
113	Coronene	191-07-1	—	**>100** (0.5 h)	*n.d.*	[47]						**Group 3**	**N.A.**
114	Cyclopenta[cd]pyrene	27208-37-3	↓	**28** (0.5 h)	*n.d.*	[47]		+	+	+		**Group 2A**	**+**
115	Dibenz[a,c]anthracene	215-58-7	**E**	**>100** or **42** (1, 0.5 h)	*n.d.*	[47,50]					**Low-moderate**	**Group 3**	**N.A.**
116	Dibenz[a,h]anthracene	53-70-3	—	**>100** (0.5, 1 h)	*n.d.*	[47,50]		+	+	+	**High**		**+**
117	Dibenz[a,j]anthracene	224-41-9	**E**	**>100** (0.5, 1 h)	*n.d.*	[47,50]					**Moderate**	**Group 3**	**N.A.**
118	Dibenzo[a,e]fluoranthene	5385-75-1	—	**>100** (0.5 h)	*n.d.*	[47]					**Marginal**	**Group 3**	**N.A.**
119	Dibenzo[a,e]pyrene	192-65-4	↓	**>100** (0.5 h)	*n.d.*	[47]					**Moderate**	**Group 3**	**+**
120	Dibenzo[a,h]pyrene	189-64-0	↓	**>100** (0.5 h)	*n.d.*	[47]					**High**	**Group 2B**	**+**
121	Dibenzo[a,i]pyrene	189-55-9	—	**>100** (0.5 h)	*n.d.*	[47]					**High**	**Group 2B**	**+**
122	Dibenzo[a,k]fluoranthene	84030-79-5	—	**>100**	*n.d.*	[47]							**N.A.**

				(0.5 h)									
123	Dibenzo[a,l]pyrene	191-30-0	↓	**>100** (0.5 h)	*n.d.*	[47]					**High**	**Group 2A**	+
124	Fluoranthene	206-44-0	↓	**9 to <70** (0.2-24 h)	**3** or **10** (80, 50 µM)	[17,26,35,43,45,47,49,51,52]						**Group 3**	+
125	Fluorene	86-73-7	↓	**38 to >90** (0.17-1.5 h)	**2** (125 µM)	[17,43,45,47,49]					**Marginal**	**Group 3**	+
126	Indeno[1,2,3-cd]pyrene	193-39-5	—	**>100** (0.5 h)	*n.d.*	[47]					**Low**	**Group 2B**	+
127	Naphthalene	91-20-3	↓	**>350** (0.07 h)	*n.d.*	[45,49]						**Group 2B**	+
128	Naphtho[2,3-a]pyrene	196-42-9	—	**>100** (0.5 h)	*n.d.*	[47]					**Low-moderate**		**N.A.**
129	Perylene	198-55-0	—	**>100** (0.5 h)	*n.d.*	[47]					**Low**	**Group 3**	+
130	Phenanthrene	85-01-8	↓	**28 to <70** (0.17-1.5 h) **>80** (24 h)	**3** (50 µM)	[17,43,45,47,49,51]					**Low**	**Group 3**	+
131	Picene	213-46-7	↓	**13** (0.5 h)	*n.d.*	[47]					**Moderate**	**Group 3**	**N.A.**
132	Pyrene	129-00-0	↓	**25 to 74** (0.2-1.5 h) **>80** (24 h)	**15** (49 µM)	[17,47,49,51,53,54]		+	—	E	**Low**	**Group 3**	+
133	Triphenylene	217-59-4	↓	**>100** (0.5 h)	*n.d.*	[47]					**Low**	**Group 3**	**N.A.**
	**PAHs chlorinated**												
134	1-Chloroanthracene	4985-70-0	↓	**45** (0.25 h)	**3** (80 µM)	[46]					**Marginal**		**N.A.**
135	2-Chloroanthracene	17135-78-3	—	**>350** (0.25 h)	*n.d.*	[46]					**Marginal**		**N.A.**
136	9-Chloroanthracene	716-53-0	↓	**42** (0.25 h)	**3** (80 µM)	[46]					**Marginal**		**N.A.**
	**PAHs methylated**												
137	10-Methylbenz[a]anthracene	2381-15-9	↓	**25** (0.5 h)	*n.d.*	[48]					**Marginal**		**N.A.**
138	11-Methylbenz[a]anthracene	6111-78-0	↓	**14** (0.5 h)	*n.d.*	[48]					**Marginal**		**N.A.**

139	12-Methylbenz[a]anthracene	2422-79-9	—	**>50** (0.5 h)	*n.d.*	[48]			Low-moderate		N.A.
140	1-Methylanthracene	610-48-0	↓	**11 to 40** (0.17-0.5 h)	**2-10** (32-75 μM)	[10,17,42–46]			Marginal		N.A.
141	1-Methylbenz[a]anthracene	2498-77-3	↓	**10** (0.5 h)	*n.d.*	[48]			Marginal		N.A.
142	1-Methylchrysene	3351-28-8	—	**>80** (1 h)	*n.d.*	[55]			Marginal	Group 3	N.A.
143	1-Methylfluorene	1730-37-6	↓	**<70 to 25** (0.2-1 h)	**2** (80 μM)	[17,43,45,49]					N.A.
144	1-Methylnaphthalene	90-12-0	↓	**200** or **<225** (0.07 h)	**0.4** or **0.9** (200 μM)	[43,45]					+
145	1-Methylpyrene	2381-21-7	↓	**24** or **<70** (0.2, 0.5 h)	*n.d.*	[17,47]			Marginal		N.A.
146	2-Methylanthracene	613-12-7	—/↓	**>400** (0.17-0.5 h)	**>480** (75 μM)	[42–46]			Marginal		N.A.
147	2-Methylbenz[a]anthracene	2498-76-2	↓	**15** (0.5 h)	*n.d.*	[48]			Low		N.A.
148	2-Methylchrysene	3351-32-4	—	**>80** (1 h)	*n.d.*	[55]			Low-moderate	Group 3	N.A.
149	2-Methylnaphthalene	91-57-6	↓	**>350** (0.07 h)	**0.8** or **0.45** (325, 320 μM)	[43,45]					N.A.
150	3-Methylbenz[a]anthracene	2498-75-1	—	**>50** (0.5 h)	*n.d.*	[48]			Marginal		N.A.
151	3-Methylchrysene	3351-31-3	—	**>80** (1 h)	*n.d.*	[55]			Low-moderate	Group 3	N.A.
152	4-Methylbenz[a]anthracene	316-49-4	—	**>50** (0.5 h)	*n.d.*	[48]			Moderate		N.A.
153	4-Methylchrysene	3351-30-2	—	**>80** (1 h)	*n.d.*	[55]			Low-moderate	Group 3	N.A.
154	5-Methylbenz[a]anthracene	2319-96-2	—	**>50** (0.5 h)	*n.d.*	[48]			Marginal		N.A.
155	5-Methylchrysene	3697-24-3	↓	**12** or **18** (0.5, 1 h)	*n.d.*	[47,55]			High	Group 2B	+
156	6-Methylbenz[a]anthracene	316-14-3	—	**>50** (0.5 h)	*n.d.*	[48]			Moderate		N.A.
157	6-Methylchrysene	1705-85-7	↓	**37** (1 h)	*n.d.*	[55]			Low-moderate	Group 3	N.A.
158	7-Methylbenz[a]anthracene	2541-69-7	—	**>50** (0.5 h)	*n.d.*	[48]			High-moderate		N.A.

159	8-Methylbenz[a]anthracene	2381-31-9	↓	**13** (0.5 h)	*n.d.*	[48]					**Moderate**		**N.A.**
160	9,10-Dimethylanthracene	781-43-1	↓	**45 to <70** (0.17 to 0.25 h)	2 to 3 (50 to 70 µM)	[17,43,45,46]					**Uncertain/ Unknown**		**N.A.**
161	9-Methylanthracene	779-02-2	↓	**26 to <50** (0.17-1.5 h)	2 to 15 (37 to 70 µM)	[42,43,45,46,49]					**Marginal**		**N.A.**
162	9-Methylbenz[a]anthracene	2381-16-0	—	**>50** (0.5 h)	*n.d.*	[48]					**Marginal**		**N.A.**
	**PAHs N-heterocyclic**												
163	Dinaphtho[2,1-b:2',3'-d]furan	204-91-1	—	**>50** (0.5 h)	*n.d.*	[56]							**N.A.**
164	7H-Dibenzo[c,g]carbazole	194-59-2	↓	**12** (0.5-24 h)	*n.d.*	[57]		**+**	**E**	**E**	**Marginal**	**Group 2B**	**+**
165	N-Methyl-7H-dibenzo[c,g]carbazole	27093-62-5	↓	**41** (0.5 h)	*n.d.*	[57]		**+**	**+**	**+**			**N.A.**
	**PAHs N-heterocyclic, methylated**												
166	5,9-Dimethyl-7H-dibenzo[c,g]carbazole	88193-04-8	↓	**39** (0.5 h)	*n.d.*	[57]		**+**	**E**	**E**			**N.A.**
	**PAHs O-heterocyclic**												
167	Dinaphtho[1,2-b:1',2'-d]furan	68518-20-7	—	**>50** (0.5 h)	*n.d.*	[56]							**N.A.**
168	Dinaphtho[1,2-b:2',1'-d]furan	239-69-0	—	**>50** (0.5 h)	*n.d.*	[56]							**N.A.**
169	Dinaphtho[1,2-b:2',3'-d]furan	239-90-7	—	**>50** (0.5 h)	*n.d.*	[56]							**N.A.**
170	Dinaphtho[2,1-b:1',2'-d]furan	194-63-8	—	**>50** (0.5 h)	*n.d.*	[56]							**N.A.**
171	Dinaphtho[2,3-b:2',3'-d]furan	242-51-3	—	**>50** (0.5 h)	*n.d.*	[56]							**N.A.**
	**PBBs**												
172	2,2',4,4',5,5'-Hexabromobiphenyl	59080-40-9	↓	**5** (12 h)	*n.d.*	[58]					**Low-moderate**		**N.A.**
	**PCBs**												
173	PCB 18	37680-65-2	↓	**10** (0.5 h)	*n.d.*	[59]					**Marginal**		**N.A.**
174	PCB 19	38444-73-4	↓	**6** (1 h)	*n.d.*	[60]					**Marginal**		**N.A.**

175	PCB 28	7012-37-5	↓	**15** or **31** (1, 0.5 h)	*n.d.*	[59,60]				Low-moderate		N.A.
176	PCB 31	16606-02-3	↓	**16** (0.5 h)	*n.d.*	[59]				Marginal		N.A.
177	PCB 47	2437-79-8	↓	**10** or **13** (0.5, 1 h)	*n.d.*	[59,60]				Low-moderate		N.A.
178	PCB 49	41464-40-8	↓	**19** (0.5 h)	*n.d.*	[59]				Low-moderate		N.A.
179	PCB 51	68194-04-7	↓	**7** (1 h)	*n.d.*	[60]				Marginal		N.A.
180	PCB 52	35693-99-3	↓	**9** or **24** (1, 0.5 h)	*n.d.*	[59,60]				Low-moderate		N.A.
181	PCB 53	41464-41-9	↓	**6** (1 h)	*n.d.*	[60]				Marginal		N.A.
182	PCB 66	32598-10-0	↓	**16** (0.5 h)	*n.d.*	[59]				Low-moderate		N.A.
183	PCB 70	32598-11-1	↓	**19** (0.5 h)	*n.d.*	[59]				Low-moderate		N.A.
184	PCB 74	32690-93-0	↓	**11** or **18** (0.5, 1 h)	*n.d.*	[59,60]				Low-moderate		N.A.
185	PCB 77	32598-13-3	—	**>100** (0.5 h)	*n.d.*	[59]				Moderate	Group 1	+
186	PCB 80	33284-52-5	—	**>50** (0.5 h)	*n.d.*	[60]				Low-moderate		N.A.
187	PCB 81	70362-50-4	—	**>100** (0.5 h)	*n.d.*	[59]				Moderate	Group 1	+
188	PCB 95	38379-99-6	↓	**8** or **17** (1, 0.5 h)	*n.d.*	[59,60]				Marginal		N.A.
189	PCB 99	38380-01-7	↓	**22** (0.5 h)	*n.d.*	[59]				Low-moderate		N.A.
190	PCB 100	39485-83-1	↓	**16** (1 h)	*n.d.*	[60]				Marginal		N.A.
191	PCB 101	37680-73-2	↓	**13** or **16** (1, 0.5 h)	*n.d.*	[59,60]				Low-moderate		N.A.
192	PCB 104	56558-16-8	↓	**8** (1 h)	*n.d.*	[60]				Marginal		N.A.
193	PCB 105	32598-14-4	↓	**21** (0.5 h)	*n.d.*	[59]				Low-moderate	Group 1	+
194	PCB 110	38380-03-9	↓	**16** (0.5 h)	*n.d.*	[59]				Low-moderate		N.A.

195	PCB 114	74472-37-0	↓	**23** (0.5 h)	*n.d.*	[59]				Low-moderate	Group 1	+
196	PCB 118	31508-00-6	↓	**17** or **19** (0.5, 1 h)	*n.d.*	[59,60]				Low-moderate	Group 1	+
197	PCB 119	56558-17-9	↓	**26** (0.5 h)	*n.d.*	[59]				Low-moderate		N.A.
198	PCB 122	76842-07-4	↓	**22** (1 h)	*n.d.*	[60]				Low-moderate		N.A.
199	PCB 123	65510-44-3	↓	**18** (0.5 h)	*n.d.*	[59]				Low-moderate	Group 1	+
200	PCB 125	74472-39-2	↓	**13** (1 h)	*n.d.*	[60]				Low-moderate		N.A.
201	PCB 126	57465-28-8	—	**>50** (0.5, 1 h)	**>2880** (50 µM)	[59,60]				High-moderate	Group 1	+
202	PCB 128	38380-07-3	↓	**13** (1 h)	*n.d.*	[60]				Marginal		N.A.
203	PCB 129	55215-18-4	↓	**23** (0.5 h)	*n.d.*	[59]				Low-moderate		N.A.
204	PCB 132	38380-05-1	↓	**18** (0.5 h)	*n.d.*	[59]				Marginal		N.A.
205	PCB 136	38411-22-2	↓	**5** or **16** (1, 0.5 h)	*n.d.*	[59,60]				Marginal		N.A.
206	PCB 138	35065-28-2	↓	**14** or **20** (1, 0.5 h)	*n.d.*	[59,60]				Low-moderate		N.A.
207	PCB 149	38380-04-0	↓	**21** (0.5 h)	*n.d.*	[59]				Marginal		N.A.
208	PCB 153	35065-27-1	↓	**15 to <50** (0.5-48 h)	*n.d.*	[17,52,59–61]				Low-moderate		+
209	PCB 156	38380-08-4	↓	**17** (0.5 h)	*n.d.*	[59]				Low-moderate	Group 1	+
210	PCB 157	69782-90-7	↓	**16** (0.5 h)	*n.d.*	[59]				Low-moderate	Group 1	+
211	PCB 163	74472-44-9	↓	**40** (0.5 h)	*n.d.*	[59]				Low-moderate		N.A.
212	PCB 167	52663-72-6	↓	**22** (0.5 h)	*n.d.*	[59]				Low-moderate	Group 1	+
213	PCB 168	59291-65-5	↓	**23** (1 h)	*n.d.*	[60]				Low-moderate		N.A.
214	PCB 169	32774-16-6	—	**>100** (0.5 h)	*n.d.*	[59]				High-moderate	Group 1	+

215	PCB 170	35065-30-6	↓	**30** or **62** (1, 0.5 h)	*n.d.*	[59,60]				**Low-moderate**		**N.A.**
216	PCB 180	35065-29-3	↓	**43** or **71** (1, 0.5 h)	*n.d.*	[59,60]				**Low-moderate**		**+**
217	PCB 187	52663-68-0	↓	**60** (0.5 h)	*n.d.*	[59]				**Marginal**		**N.A.**
218	PCB 189	39635-31-9	↓	**>100** (0.5 h)	*n.d.*	[59]				**Low-moderate**	**Group 1**	**+**
219	PCB 190	41411-64-7	↓	**23** (1 h)	*n.d.*	[60]				**Low-moderate**		**N.A.**
220	PCB 194	35694-08-7	—	**>100** (0.5 h)	*n.d.*	[59]				**Low-moderate**		**N.A.**
	**PCBs metabolites**											
221	2-(2′,4′,5′-tri-Cl-phenyl)-1,4-Hydroquinone		↓	**>100** (0.5 h)	*n.d.*	[62]						
222	2-(2′-Cl-phenyl)-1,4-Benzoquinones		↓	**>100** (0.5 h)	*n.d.*	[62]						
223	2-(2′-Cl-phenyl)-1,4-Hydroquinone		—	**>100** (0.5 h)	*n.d.*	[62]						
224	2-(3′,4′,5′-tri-Cl-phenyl)-1,4-Benzoquinones		↓	**42** (0.5 h)	*n.d.*	[62]						
225	2-(3′,4′,5′-tri-Cl-phenyl)-1,4-Hydroquinone		↓	**>100** (0.5 h)	*n.d.*	[62]						
226	2-(3′,4′-di-Cl-phenyl)-1,4-Benzoquinones		↓	**49** (0.5 h)	*n.d.*	[62]						
227	2-(3′,4′-di-Cl-phenyl)-1,4-Hydroquinone		↓	**>100** (0.5 h)	*n.d.*	[62]						
228	2-(3′,5′-di-Cl-phenyl)-1,4-Benzoquinones		↓	**37** (0.5 h)	*n.d.*	[62]						
229	2-(3′,5′-di-Cl-phenyl)-1,4-Hydroquinone		—	**>100** (0.5 h)	*n.d.*	[62]						
230	2-(3′-Cl-phenyl)-1,4-Benzoquinones		↓	**70** (0.5 h)	*n.d.*	[62]						
231	2-(3′-Cl-phenyl)-1,4-Hydroquinone		—	**>100** (0.5 h)	*n.d.*	[62]						
232	2-(4′-Cl-phenyl)-1,4-Benzoquinones		↓	**73** (0.5 h)	*n.d.*	[62]						
233	2-(4′-Cl-phenyl)-1,4-Hydroquinone		↓	**>100** (0.5 h)	*n.d.*	[62]						
234	2,2′-Biphenyldiol	1806-29-7	↓	**225**	**45**	[63]						**N.A.**

				(0.5 h)	(250 µM)							
235	2′-Hydroxy-PCB 3	64181-76-6	—	**>100** (0.5 h)	*n.d.*	[62]				**Low-moderate**		
236	2-Biphenylol	90-43-7	↓	**>300** (0.5 h)	**480** (230 µM)	[63]					**Group 3**	**+**
237	3,3′-Biphenyldiol	612-76-0	↓	**>300** (0.5 h)	**1440** (250 µM)	[63]						
238	3′,4′-Dihydroxy-PCB 3		↓	**43** (0.5 h)	*n.d.*	[62]						
239	3′,4′-Dihydroxy-PCB 5		↓	**16** (0.5 h)	*n.d.*	[62]						
240	3′,4′-Dihydroxy-PCB 12		↓	**19** (0.5 h)	*n.d.*	[62]						
241	3′-Hydroxy-PCB 3		↓	**37** (0.5 h)	*n.d.*	[62]						
242	3-Biphenylol	580-51-8	↓	**>300** (0.5 h)	**1440** (150 µM)	[63]						**N.A.**
243	3-Chloro-2-biphenylol	85-97-2	↓	**>300** (0.5 h)	**>1440** (100 µM)	[63]				**Marginal**		**N.A.**
244	4,4′-Dichloro-3-biphenylol	53459-39-5	↓	**30** (0.5 h)	**10** (40 µM)	[63]				**Low-moderate**		**N.A.**
245	4′-Hydroxy-PCB 3		↓	**31** (0.5 h)	*n.d.*	[62]						
246	4′-Hydroxy-PCB 36		↓	**38** (0.5 h)	*n.d.*	[62]						
247	4′-Hydroxy-PCB 68		↓	**22** (0.5 h)	*n.d.*	[62]						
248	4′-Hydroxy-PCB 79		↓	**27** (0.5 h)	*n.d.*	[62]						
249	4-Hydroxy-PCB 14		↓	**38** (0.5 h)	*n.d.*	[62]						
250	4-Hydroxy-PCB 34		↓	**32** (0.5 h)	*n.d.*	[62]						
251	4-Hydroxy-PCB 35		↓	**31** (0.5 h)	*n.d.*	[62]						
252	4-Hydroxy-PCB 36		↓	**30** (0.5 h)	*n.d.*	[62]						
253	4-Hydroxy-PCB 39		↓	**27** (0.5 h)	*n.d.*	[62]						
254	4-Hydroxy-PCB 107	152969-11-4	↓	**20**	*n.d.*	[62]				**Marginal**		**N.A.**

				(0.5 h)									
255	4-Hydroxy-PCB 187	158076-68-7	↓	**9** (0.5 h)	*n.d.*	[62]					Low		N.A.
256	4-Hydroxy-PCB-146		↓	**13** (0.5 h)	*n.d.*	[62]							
257	6′-Hydroxy-PCB 35		↓	**24** (0.5 h)	*n.d.*	[62]							
258	6′-Hydroxy-PCB 36		↓	**20** (0.5 h)	*n.d.*	[62]							
	**PCDDs**												
259	TCDD	1746-01-6	↓	**>0.001** (24 h)	**>48** (0.001 µM)	[26,64]	—				**High**	**Group 1**	+
	**Peptides**												
260	Cylindrospermopsin	143545-90-8	—	**>60** (0.5 h)	*n.d.*	[52]							N.A.
261	EGF	62229-50-9	↓	**<0.001 to <0.008** (0.2-0.5 h)	**2** (0.002 µM)	[17,29,59,65,66]	—						N.A.
262	Microcystin-LR	101043-37-2	—	**>100** (0.5 h)	*n.d.*	[52]						**Group 2B**	+
263	TNFα	308079-78-9	↓	**<0.0003** (24 h)	*n.d.*	[51]							N.A.
264	TRAP 6	141136-83-6	↓	**<50** (0.5 h)	*n.d.*	[17]							N.A.
	**Peroxides**												
265	Hydrogen peroxide H_2O_2	7722-84-1	↓	**<100 to >1000** (0.5-1 h)	*n.d.*	[15,20,67–81]	—	+	+	+		**Group 3**	+
	**PFASs**												
266	Perfluorobutanesulfonic acid PFBS	375-73-5	—	**>160** (0.3 h)	*n.d.*	[82]							N.A.
267	Perfluorobutanoic acid PFBA	375-22-4	—	**>300** (0.3 h)	*n.d.*	[21]							N.A.
268	Perfluorodecanoic acid PFDA	335-76-2	↓	**25** or **<50** (0.3 h)	**2** (350 µM)	[17,21]					**Low-moderate**		+
269	Perfluoroheptanoic acid PFHpA	375-85-9	↓	**140** (0.3 h)	*n.d.*	[21]							N.A.
270	Perfluorohexadecanoic acid PFHA	67905-19-5	—	**>300** (0.3 h)	*n.d.*	[21]							N.A.

271	Perfluorohexanesulfonic acid PFHS	355-46-4	↓	**122** (0.5 h)	*n.d.*	[82]							**N.A.**
272	Perfluorononanoic acid PFNA	375-95-1	↓	**90** (0.3 h)	*n.d.*	[21]							**N.A.**
273	Perfluorooctadecanoic acid PFODA	16517-11-6	—	**>300** (0.3 h)	*n.d.*	[21]							**N.A.**
274	Perfluorooctane sulfonate PFOS	1763-23-1	↓	**30** or **<40** (0.5, 0.3 h)	**2** (50 µM)	[17,82]							**N.A.**
275	Perfluorooctanesulfonamide PFOSA	754-91-6	↓	**25** or **37** (0.3, 0.5 h)	**2** or **10** (50, 350 µM)	[21]							**N.A.**
276	Perfluorooctanoic acid PFOA	335-67-1	↓	**50 to 100** (0.08-0.3 h)	**2** (350 µM)	[17,21,35,83]						**Group 2B**	+
277	Perfluoropentanoic acid PFPeA	2706-90-3	—	**>200** (0.3 h)	*n.d.*	[21]							**N.A.**
278	Perfluoropropanoic acid PFPA	422-64-0	—	**>300** (0.3 h)	*n.d.*	[21]							**N.A.**
279	Trifluoroacetic acid TFA	76-05-1	—	**>300** (0.3 h)	*n.d.*	[21]							**N.A.**
	**Phenothiazines**												
280	Mesoridazine	5588-33-0	↓	**5** (1 h)	*n.d.*	[37]							**N.A.**
	**Phorbols**												
281	TPA	16561-29-8	↓	**0.002 to 0.02** (0-1 h)	**<10** (0.016 µM)	[15,17,62,64,66,68,77,84–88,35,89,90,36,47,55–57,59,61]	↓						+
	**Phthalates**												
282	Benzyl butyl phthalate BBP	85-68-7	↓	**21** (0.5 h)	**2** (80 µM)	[91]			+	+			+
283	Di(2-ethylhexyl) phthalate DEHP	117-81-7	↓	**>200** (0.5 h)	**207** (80 µM)	[91]	E	—	—	—		**Group 2B**	+
284	Diallyl phthalate DAP	131-17-9	↓	**100** (0.5 h)	**>1440** (80 µM)	[91]		—	+	E			**N.A.**
285	Dibutyl phthalate DBP	84-74-2	↓	**13** (0.5 h)	**2** (80 µM)	[91]							+
286	Dicyclohexyl phthalate DCHP	84-61-7	↓	**22** (0.5 h)	**10** (80 µM)	[91]							**N.A.**
287	Didecyl phthalate DDP	84-77-5	—	**>200** (0.5 h)	**>1440** (80 µM)	[91]							**N.A.**
288	Diethyl phthalate DEP	84-66-2	—	**>200**	**>1440**	[91]	—						+

				(0.5 h)	(80 μM)							
289	Diheptyl phthalate DHpP	3648-21-3	↓	**58** (0.5 h)	**25** (80 μM)	[91]						+
290	Diisibutyl phthalate DIBP	84-69-5	↓	**17** (0.5 h)	**10** (80 μM)	[91]						
291	Diisodecyl phthalate DIDP	26761-40-0	—	**>200** (0.5 h)	**>1440** (80 μM)	[91]						**N.A.**
292	Diisoheptyl phthalate DIHpP	41451-28-9	↓	**44** (0.5 h)	**17** (80 μM)	[91]						**N.A.**
293	Diisononyl phthalate DINP	28553-12-0	↓	**>200** (0.5 h)	**711** (80 μM)	[91]						**N.A.**
294	Diisopropyl phthalate DIPrP	605-45-8	↓	**86** (0.5 h)	**>1440** (80 μM)	[91]						**N.A.**
295	Dimethyl phthalate DMP	131-11-3	—	**>200** (0.5 h)	**>1440** (80 μM)	[91]						+
296	Dioctyl phthalate DOP	117-84-0	↓	**>200** (0.5 h)	**292** (80 μM)	[91]						+
297	Dipentyl phthalate DPeP	131-18-0	↓	**16** (0.5 h)	**10** (80 μM)	[91]						**N.A.**
298	Diphenyl phthalate DPhP	84-62-8	↓	**39** (0.5 h)	**10** (80 μM)	[91]						**N.A.**
299	Dipropyl phthalate DPrP	131-16-8	↓	**39** (0.5 h)	**<10** (80 μM)	[91]						**N.A.**
300	Monobutyl phthalate MBP	131-70-4	—	**>200** (0.5 h)	**>1440** (80 μM)	[91]						**N.A.**
301	Monomethyl phthalate MMP	4376-18-5	—	**>200** (0.5 h)	**>1440** (80 μM)	[91]						**N.A.**
	**Piperidines**											
302	Thioridazine	50-52-2	↓	**5** (1 h)	**5** (10 μM)	[37]						**N.A.**
	**Platinum compounds**											
303	Cisplatin	15663-27-1	—	**>10** (0-8 h)	**>480** (10 μM)	[90]		+	+	+	**Moderate**	+
304	Platinum complex LA-12	250611-20-2	↓	**1** or **1.5** (2, 3 h)	**40** or **60** (10, 8 μM)	[90]						**N.A.**
	**Polyphenolic compounds**											
305	Gossypol	303-45-7	↓	**<3** (24 h)	*n.d.*	[92]	—				**Low to Moderate**	**N.A.**

									**to Marginal**	
	**Polysaccharides**									
306	Lambda-Carrageenan (extract)		↓	**0.00005%** (0.5 h)	**10** (0.001%)	[93]				
	**Pyrene ozonation byproducts**									
307	1,2,3,4-Tetrahydro-9-phenanthrene carboxaldehyde		↓	**25** (0.5 h)	**15** (*n.s.*)	[54]				
308	2-Biphenyl carboxylic acid	947-84-2	↓	**>200** (0.5 h)	*n.d.*	[54]				**N.A.**
309	4-Biphenyl carboxaldehyde	3218-36-8	↓	**110** (0.5 h)	**15** (*n.s.*)	[54]			**Low-moderate to Moderate**	**N.A.**
310	4-Biphenyl carboxylic acid	92-92-2	↓	**>200** (0.5 h)	*n.d.*	[54]				**N.A.**
311	4-Carboxy-5-phenanthrene (4C5P)		↓	**>100** (0.5 h)	*n.d.*	[54]				
312	4-Carboxy-phenanthrene (4CP)		↓	**>100** (0.5 h)	*n.d.*	[54]				
313	9-Oxo-1-fluorene carboxaldehyde		↓	**25** (0.5 h)	**15** (*n.s.*)	[54]				
314	Biphenyl-2,2',6,6'-tetracarbaldehyde	4371-26-0	↓	**23** (0.5 h)	**60** (5 µM)	[53]				**N.A.**
315	Biphenyl-2,2',6,6'-tetracarboxylic acid	4371-27-1	—	**>100** (0.5 h)	**>360** (500 µM)	[53]				**N.A.**
316	Diphenic acid	482-05-3	↓	**>200** (0.5 h)	*n.d.*	[54]				**N.A.**
	**Retinoids**									
317	11-cis-Retinaldehyde	564-87-4	↓	**<10** (0.5 h)	*n.d.*	[9]				**N.A.**
318	All-trans-Retinaldehyde	116-31-4	↓	**2.5** (0.5 h)	*n.d.*	[9]			**Low**	**N.A.**
319	All-trans-Retinoic acid	302-79-4	↓	**30** (0.5 h)	*n.d.*	[9]	↓			+
	**Sphingolipids**									
320	C2-Ceramide	3102-57-6	↓	**>40** (0-4 h)	**>240** (40 µM)	[94]				**N.A.**
321	C6-Ceramide	124753-97-5	↓	**15**	**7**	[94]				**N.A.**

				(0.5 h)	(40 µM)								
322	C8-Ceramide	74713-59-0	↓	**15** (0.5 h)	**15** (40 µM)	[94]							
	**Steroids**												
323	17β-Estradiol	50-28-2	↓	**>250** (24 h)	*n.d.*	[10]	—	—	E	E		**Group 1 ***	+
324	Fulvestrant	129453-61-8	—	**>250** (0.5-24 h)	**>1440** (250 µM)	[10]							**N.A.**
325	Testosterone	58-22-0	—	**>250** (0.5-24 h)	**>1440** (1-250 µM)	[10]	E						+
	**Trihydroxybenzoic acids**												
326	Gallic acid	149-91-7	↓	**<100** (0.5, 1 h)	*n.d.*	[25,76,78]							**N.A.**
	**Triterpenoids**												
327	18α-glycyrrhetinic acid	1449-05-4	↓	**65** (0.5 h)	*n.d.*	[9]	↓						**N.A.**
328	18β-glycyrrhetinic acid	471-53-4	↓	**<30** (0.3 h)	*n.d.*	[17]	↓						**N.A.**

CASRN, Chemical Abstracts Service Registry Number; **DDT**, dichlorodiphenyltrichloroethane; **EC**$_{50}$, the concentration causing 50% GJIC inhibition; **EGF**, epidermal growth factor; **ET**$_{50}$, the exposure time causing 50% GJIC inhibition; **GJIC**, gap junctional intercellular communication; **MBOCA**, 4,4'-methylene bis (2-chloroaniline); **MC**, metabolic cooperation; **NA**, not available; ***n.d.***, not determined; ***n.s.***, not specified; **PAHs**, polycyclic aromatic hydrocarbons; **PBBs**, polybrominated biphenyls; **PCBs**, polychlorinated biphenyls; **PCDDs**, polychlorinated dibenzo-p-dioxins; **PFASs**, perfluoroalkyl and polyfluoroalkyl substances; **Ref.**, reference; **TCDD**, tetrachlorodibenzo-p-dioxin; **THC**, delta-9-tetrahydrocannabinol; **TNFα**, tumor necrosis factor-alpha; **TRAP 6**, thrombin receptor activator peptide 6; **SL-DT**, scrape loading-dye transfer
*(oestrogen-only menopausal therapy)

Legend:

SL-DT assay

↓	Significant GJIC inhibition and the EC_{50} value could be calculated
↓	Significant GJIC inhibition, but less then 50% of a negative control (the EC_{50} value could not be calculated)
–	No GJIC inhibition
–/↓	More studies report no GJIC inhibition than significant GJIC inhibition
E	Equivocal result (no GJIC inhibition:significant GJIC inhibition = 1:1)

Genotoxicity

+	Positive
–	Negative
E	Equivocal result, when response is weak or not reproduced between experiments or between laboratories
I	Inconclusive or (more usually) inadequately tested (e.g. not tested both - and + S9, insufficient concentrations, insufficient toxicity etc.)

IARC

**Group 1**	"Carcinogenic to humans" There is enough evidence to conclude that it can cause cancer in humans.
**Group 2A**	"Probably carcinogenic to humans" There is strong evidence that it can cause cancer in humans, but at present it is not conclusive.
**Group 2B**	"Possibly carcinogenic to humans" There is some evidence that it can cause cancer in humans but at present it is far from conclusive.
**Group 3**	"Unclassifiable as to carcinogenicity in humans" There is no evidence at present that it causes cancer in humans.

CompTox/ToxRefDB

+	Positive
NA	Not available

MC assay

↓	GJIC inhibition
–	No GJIC inhibition
E	Equivocal result / Uncertain

OncoLogic

**Low**	Unlikely to be a carcinogen
**Marginal**	Likely to have equivocal carcinogenic activity
**Low-moderate**	Likely to be weakly carcinogenic
**Moderate**	Likely to be moderately active carcinogen
**High-moderate**	Highly likely to be a moderately active carcinogen
**High**	Highly likely to be a potent carcinogen
**Low-moderate to Moderate**	Likely to be weakly carcinogenic to Likely to be moderately active carcinogen
**Uncertain/ Unknown**	Uncertain
**Marginal to High-moderate**	Uncertain
**Marginal to Low-moderate**	Uncertain
**Low to Moderate to Marginal**	Uncertain
**Low to Moderate (High-moderate)**	Uncertain

Supplementary Figures

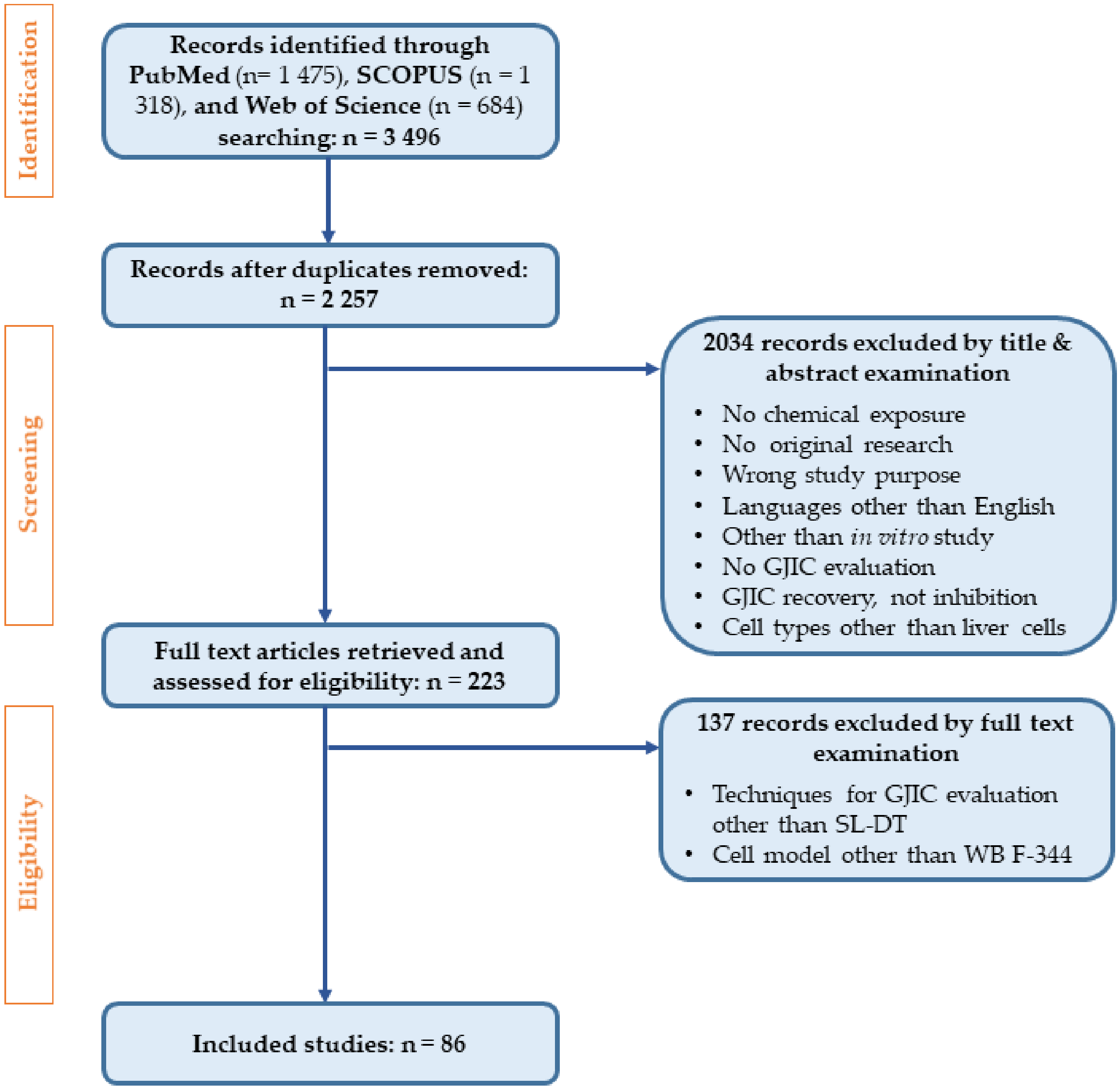

Supplementary Figure S1. A study flow for the systematic literature search and exclusion criteria.

References

1. Sakai, A.; Iwase, Y.; Nakamura, Y.; Sasaki, K.; Tanaka, N.; Umeda, M. Use of a cell transformation assay with established cell lines, and a metabolic cooperation assay with V79 cells for the detection of tumour promoters: a review. *Altern. Lab. Anim.* **2002**, *30*, 33–59, doi:10.1177/026119290203000105.

2. Rosenkranz, M.; Rosenkranz, H.S.; Klopman, G. Intercellular communication, tumor promotion and non-genotoxic carcinogenesis: relationships based upon structural considerations. *Mutat. Res.* **1997**, *381*, 171–188, doi:10.1016/s0027-5107(97)00165-6.

3. Corvi, R.; Madia, F. EURL ECVAM genotoxicity and carcinogenicity consolidated database of Ames positive chemicals. *Eur. Comm. Jt. Res. Cent. [Dataset] PID* **2018**.

4. Madia, F.; Kirkland, D.; Morita, T.; White, P.; Asturiol, D.; Corvi, R. EURL ECVAM Genotoxicity and Carcinogenicity Database of Substances Eliciting Negative Results in the Ames Test: Construction of the Database. *Mutat. Res. Toxicol. Environ. Mutagen.* **2020**, *854–855*, 503199, doi:https://doi.org/10.1016/j.mrgentox.2020.503199.

5. Williams, A.J.; Grulke, C.M.; Edwards, J.; McEachran, A.D.; Mansouri, K.; Baker, N.C.; Patlewicz, G.; Shah, I.; Wambaugh, J.F.; Judson, R.S.; et al. The CompTox Chemistry Dashboard: a community data resource for environmental chemistry. *J. Cheminform.* **2017**, *9*, 61, doi:10.1186/s13321-017-0247-6.

6. US EPA Oncologic 9/8 Available online: https://www.epa.gov/tsca-screening-tools/oncologictm-expert-system-evaluate-carcinogenic-potential-chemicals.

7. IARC No Title Available online: https://monographs.iarc.fr/list-of-classifications (accessed on Oct 10, 2020).

8. Watford, S.; Ly Pham, L.; Wignall, J.; Shin, R.; Martin, M.T.; Friedman, K.P. ToxRefDB version 2.0: Improved utility for predictive and retrospective toxicology analyses. *Reprod. Toxicol.* **2019**, *89*, 145–158, doi:https://doi.org/10.1016/j.reprotox.2019.07.012.

9. Pulukuri, S.; Sitaramayya, A. Retinaldehyde, a potent inhibitor of gap junctional intercellular communication. *Cell Commun. Adhes.* **2004**, *11*, 25–33, doi:10.1080/15419060490471784.

10. Babica, P.; Zurabian, R.; Kumar, E.R.; Chopra, R.; Mianecki, M.J.; Park, J.-S.; Jasa, L.; Trosko, J.E.; Upham, B.L. Methoxychlor and Vinclozolin Induce Rapid Changes in Intercellular and Intracellular Signaling in Liver Progenitor Cells. *Toxicol. Sci.* **2016**, *153*, 174–185, doi:10.1093/toxsci/kfw114.

11. Kuslikis, B.I.; Trosko, J.E.; Braselton, W.E.J. Mutagenicity and effect on gap-junctional intercellular communication of 4,4′-methylenebis(2-chloroaniline) and its oxidized metabolites. *Mutagenesis* **1991**, *6*, 19–24, doi:10.1093/mutage/6.1.19.

12. Kang, N.J.; Lee, K.M.; Kim, J.H.; Lee, B.K.; Kwon, J.Y.; Lee, K.W.; Lee, H.J. Inhibition of gap junctional intercellular communication by the green tea polyphenol (-)-epigallocatechin gallate in normal rat liver epithelial cells. *J. Agric. Food Chem.* **2008**, *56*, 10422–10427, doi:10.1021/jf801981w.

13. Upham, B.L.; Rummel, A.M.; Carbone, J.M.; Trosko, J.E.; Ouyang, Y.; Crawford, R.B.; Kaminski, N.E. Cannabinoids inhibit gap junctional intercellular communication and activate ERK in a rat liver epithelial cell line. *Int. J. cancer* **2003**, *104*, 12–18, doi:10.1002/ijc.10899.

14. Upham, B.L.; Boddy, B.; Xing, X.S.; Trosko, J.E.; Masten, S.J. Non-genotoxic effects of selected pesticides and their disinfection by-products on gap junctional intercellular communication. *OZONE-SCIENCE Eng.* **1997**, *19*, 351–369, doi:10.1080/01919519708547298.

15. Kang, K.S.; Kang, B.C.; Lee, B.J.; Che, J.H.; Li, G.X.; Trosko, J.E.; Lee, Y.S. Preventive effect of epicatechin and ginsenoside Rb(2) on the inhibition of gap junctional intercellular communication by TPA and H(2)O(2). *Cancer Lett.* **2000**, *152*, 97–106, doi:10.1016/s0304-3835(99)00438-3.

16. Abdelmohsen, K.; Stuhlmann, D.; Daubrawa, F.; Klotz, L.-O.L.-O.L.-O. Dicumarol is a potent reversible inhibitor of gap junctional intercellular communication. *Arch. Biochem. Biophys.* **2005**, *434*, 241–247, doi:10.1016/j.abb.2004.11.002.

17. Sovadinova, I.; Babica, P.; Boke, H.; Kumar, E.; Wilke, A.; Park, J.-S.; Trosko, J.E.; Upham, B.L.; Raza, F.A.; Rehman, S.U.; et al. Phosphatidylcholine Specific PLC-Induced Dysregulation of Gap Junctions, a Robust Cellular Response to Environmental Toxicants, and Prevention by Resveratrol in a Rat Liver Cell Model. *PLoS One* **2015**, *10*, e0124454, doi:10.1371/journal.pone.0137599.

18. Fransson, R.; Nicotera, P.; Wärngård, L.; Ahlborg, U.G. Changes in cytosolic CA2+ are not involved in DDT-induced loss of gap junctional communication in WB-F344 cells. *Cell Biol. Toxicol.* **1990**, *6*, 235–244, doi:10.1007/BF00249596.

19. Hii, C.S.; Ferrante, A.; Schmidt, S.; Rathjen, D.A.; Robinson, B.S.; Poulos, A.; Murray, A.W. Inhibition of gap junctional communication by polyunsaturated fatty acids in WB cells: evidence that connexin 43 is not hyperphosphorylated. *Carcinogenesis* **1995**, *16*, 1505–1511, doi:10.1093/carcin/16.7.1505.

20. Lee, K.M.; Kwon, J.Y.; Lee, K.W.; Lee, H.J. Ascorbic acid 6-palmitate suppresses gap-junctional intercellular communication through phosphorylation of connexin 43 via activation of the MEK-ERK pathway. *Mutat. Res. Mol. Mech. Mutagen.* **2009**, *660*, 51–56, doi:10.1016/j.mrfmmm.2008.10.012.

21. Upham, B.L.; Deocampo, N.D.; Wurl, B.; Trosko, J.E. Inhibition of gap junctional intercellular communication by perfluorinated fatty acids is dependent on the chain length of the fluorinated tail. *Int. J. CANCER* **1998**, *78*, 491–495, doi:10.1002/(SICI)1097-0215(19981109)78:4<491::AID-IJC16>3.0.CO;2-9.

22. Hakulinen, P.; Rintala, E.; Mäki-Paakkanen, J.; Komulainen, H. Altered expression of connexin43 in the inhibition of gap junctional intercellular communication by chlorohydroxyfuranones in WB-F344 rat liver epithelial cells. *Toxicol. Appl. Pharmacol.* **2006**, *212*, 146–155, doi:10.1016/j.taap.2005.07.013.

23. Flodström, S.; Warngård, L.; Hemming, H.; Ahlborg, U.G. Chlorobenzilate-induced effects on enzyme-altered foci in rat liver and intercellular communication in rat liver WB-F344 epithelial cells. *Cancer Lett.* **1988**, *43*, 161–166, doi:10.1016/0304-3835(88)90165-6.

24. Sai, K.; Upham, B.L.; Kang, K.S.; Hasegawa, R.; Inoue, T.; Trosko, J.E. Inhibitory effect of pentachlorophenol on gap junctional intercellular communication in rat liver epithelial cells in vitro. *Cancer Lett.* **1998**, *130*, 9–17, doi:10.1016/s0304-3835(98)00082-2.

25. Kim, J.H.; Kang, N.J.; Lee, B.K.; Lee, K.W.; Lee, H.J. Gallic acid, a metabolite of the antioxidant propyl gallate, inhibits gap junctional intercellular communication via phosphorylation of connexin 43 and extracellular-signal-regulated kinase1/2 in rat liver epithelial cells. *Mutat. Res.* **2008**, *638*, 175–183, doi:10.1016/j.mrfmmm.2007.10.005.

26. Forejtníková, H.; Lunerová, K.; Kubínová, R.; Jankovská, D.; Marek, R.; Kares, R.; Suchý, V.; Vondrácek, J.; Machala, M. Chemoprotective and toxic potentials of synthetic and natural chalcones and dihydrochalcones in vitro. *Toxicology* **2005**, *208*, 81–93, doi:10.1016/j.tox.2004.11.011.

27. Nishikawa, A.; Sai, K.; Okazaki, K.; Son, H.-Y.; Kanki, K.; Nakajima, M.; Kinae, N.; Nohmi, T.; Trosko, J.E.; Inoue, T.; et al. MX, a by-product of water chlorination, lacks in vivo genotoxicity in gpt delta mice but inhibits gap junctional intercellular communication in rat WB cells. *Environ. Mol. Mutagen.* **2006**, *47*, 48–55, doi:10.1002/em.20167.

28. Hsiao, P.-J.; Jao, J.-C.; Tsai, J.-L.; Chang, W.-T.; Jeng, K.-S.; Kuo, K.-K. Inorganic arsenic trioxide induces gap junction loss in association with the downregulation of connexin43 and E-cadherin in rat hepatic “stem-like” cells. *Kaohsiung J. Med. Sci.* **2014**, *30*, 57–67, doi:10.1016/j.kjms.2013.10.002.

29. Hill, C.S.; Oh, S.Y.; Schmidt, S.A.; Clark, K.J.; Murray, A.W. Lysophosphatidic acid inhibits gap-junctional communication and stimulates phosphorylation of connexin-43 in WB cells: possible involvement of the mitogen-activated protein kinase cascade. *Biochem. J.* **1994**, *303 (Pt 2*, 475–479, doi:10.1042/bj3030475.

30. Jeong, S.H.; Cho, M.H.; Cho, J.H.; Jeon, S.H.; Cho, M.H.; Cho, J.H.; Jeong, S.H.; Cho, M.H.; Cho, J.H.; Jeon, S.H.; et al. Effects of cadmium on gap junctional intercellular communication in WB-F344 rat liver epithelial cells. *Hum. Exp. Toxicol.* **2001**, *20*, 577–583, doi:10.1191/096032701718620855.

31. Masten, S.J.; Tian, M.; Upham, B.L.; Trosko, J.E. Effect of selected pesticides and their ozonation by-products on gap junctional intercellular communication using rat liver epithelial cell lines. *Chemosphere* **2001**, *44*, 457–465, doi:10.1016/s0045-6535(00)00296-4.

32. Alpatova, A.L.; Shan, W.; Babica, P.; Upham, B.L.; Rogensues, A.R.; Masten, S.J.; Drown, E.; Mohanty, A.K.; Alocilja, E.C.; Tarabara, V. V Single-walled carbon nanotubes dispersed in aqueous media via non-covalent functionalization: effect of dispersant on the stability, cytotoxicity, and epigenetic toxicity of nanotube suspensions. *Water Res.* **2010**, *44*, 505–520, doi:10.1016/j.watres.2009.09.042.

33. Upham, B.L.; Guzvić, M.; Scott, J.; Carbone, J.M.; Blaha, L.; Coe, C.; Li, L.L.; Rummel, A.M.; Trosko, J.E. Inhibition of gap junctional intercellular communication and activation of mitogen-activated protein kinase by tumor-promoting organic peroxides and protection by resveratrol. *Nutr. Cancer* **2007**, *57*, 38–47, doi:10.1080/01635580701268188.

34. Flodström, S.; Hemming, H.; Wärngård, L.; Ahlborg, U.G. Promotion of altered hepatic foci development in rat liver, cytochrome P450 enzyme induction and inhibition of cell-cell communication by DDT and some structurally related organohalogen pesticides. *Carcinogenesis* **1990**, *11*, 1413–1417, doi:10.1093/carcin/11.8.1413.

35. Babica, P.; Čtveráčková, L.; Lenčešová, Z.; Trosko, J.E.; Upham, B.L. Chemopreventive Agents Attenuate Rapid Inhibition of Gap Junctional Intercellular Communication Induced by Environmental Toxicants. *Nutr. Cancer* **2016**, *68*, 827–837, doi:10.1080/01635581.2016.1180409.

36. Nielsen, M.; Ruch, R.J.; Vang, O. Resveratrol reverses tumor-promoter-induced inhibition of gap-junctional intercellular communication. *Biochem. Biophys. Res. Commun.* **2000**, *275*, 804–809, doi:10.1006/bbrc.2000.3378.

37. Matesic, D.F.; Abifadel, D.N.; Garcia, E.L.; Jann, M.W. Effect of thioridazine on gap junction intercellular communication in connexin 43-expressing cells. *Cell Biol. Toxicol.* **2006**, *22*, 257–268, doi:10.1007/s10565-006-0047-7.

38. Horvath, A.; Upham, B.L.; Ganev, V.; Trosko, J.E. Determination of the epigenetic effects of ochratoxin in a human kidney and a rat liver epithelial cell line. *Toxicon* **2002**, *40*, 273–282, doi:10.1016/s0041-0101(01)00219-7.

39. Lee, C.-H.; Chen, I.-H.; Lee, C.-R.; Chi, C.-H.; Tsai, M.-C.; Tsai, J.-L.; Lin, H.-F. Inhibition of gap junctional Intercellular communication in WB-F344 rat liver epithelial cells by triphenyltin chloride through MAPK and PI3-kinase pathways. *J. Occup. Med. Toxicol.* **2010**, *5*, 17, doi:10.1186/1745-6673-5-17.

40. Sharov, V.S.; Briviba, K.; Sies, H. Peroxynitrite diminishes gap junctional communication: protection by selenite supplementation. *IUBMB Life* **1999**, *48*, 379–384, doi:10.1080/713803538.

41. Lee, B.K.; Chung, M.-Y.; Lee, K.W. Benzo[a]pyrene-7,8-diol-9,10-epoxide inhibits gap junction intercellular communication via phosphorylation of tumor progression locus 2 in WB-F344 rat liver epithelial cells. *Mol. Carcinog.* **2015**, *54*, 351–358, doi:10.1002/mc.22103.

42. Upham, B.L.; Weis, L.M.; Rummel, A.M.; Masten, S.J.; Trosko, J.E. The effects of anthracene and methylated anthracenes on gap junctional intercellular communication in rat liver epithelial cells. *Fundam. Appl. Toxicol.* **1996**, *34*, 260–264, doi:10.1006/faat.1996.0195.

43. Upham, B.L.; Weis, L.M.; Trosko, J.E. Modulated gap junctional intercellular communication as a biomarker of PAH epigenetic toxicity: structure-function relationship. *Environ. Health Perspect.* **1998**, *106 Suppl*, 975–981, doi:10.1289/ehp.98106s4975.

44. Upham, B.L.; Blaha, L.; Babica, P.; Park, J.-S.; Sovadinova, I.; Pudrith, C.; Rummel, A.M.; Weis, L.M.; Sai, K.; Tithof, P.K.; et al. Tumor promoting properties of a cigarette smoke prevalent polycyclic aromatic hydrocarbon as indicated by the inhibition of gap junctional intercellular communication via phosphatidylcholine-specific phospholipase C. *Cancer Sci.* **2008**, *99*, 696–705, doi:10.1111/j.1349-7006.2008.00752.x.

45. Weis, L.M.; Rummel, A.M.; Masten, S.J.; Trosko, J.E.; Upham, B.L. Bay or baylike regions of polycyclic aromatic hydrocarbons were potent inhibitors of gap junctional intercellular communication. *Environ. Health Perspect.* **1998**, *106*, 17–22, doi:10.1289/ehp.9810617.

46. Rummel, A.M.; Trosko, J.E.; Wilson, M.R.; Upham, B.L. Polycyclic aromatic hydrocarbons with bay-like regions inhibited gap junctional intercellular communication and stimulated MAPK activity. *Toxicol. Sci.* **1999**, *49*, 232–240, doi:10.1093/toxsci/49.2.232.

47. Bláha, L.; Kapplová, P.; Vondráček, J.; Upham, B.; Machala, M.; Vondrácek, J.; Upham, B.; Machala, M.; Vondráček,

J.; Upham, B.; et al. Inhibition of gap-junctional intercellular communication by environmentally occuring polycyclic aromatic hydrocarbons. *Toxicol. Sci.* **2002**, *65*, 43–51, doi:10.1093/toxsci/65.1.43.

48. Marvanová, S.; Vondrácek, J.; Penccíková, K.; Trilecová, L.; Krcmárr, P.; Topinka, J.; Nováková, Z.; Milcová, A.; Machala, M. Toxic effects of methylated benz[a]anthracenes in liver cells. *Chem. Res. Toxicol.* **2008**, *21*, 503–512, doi:10.1021/tx700305x.

49. Upham, B.L.; Masten, S.J.; Lockwood, B.R.; Trosko, J.E. Nongenotoxic effects of polycyclic aromatic hydrocarbons and their oxygenation by-products on the intercellular communication of rat liver epithelial cells. *Fundam. Appl. Toxicol.* **1994**, *23*, 470–475, doi:10.1006/faat.1994.1129.

50. Svihálková-Sindlerová, L.; Machala, M.; Pencíková, K.; Marvanová, S.; Neca, J.; Topinka, J.; Sevastyanova, O.; Kozubík, A.; Vondrácek, J.; Švihálková-Šindlerová, L.; et al. Dibenzanthracenes and benzochrysenes elicit both genotoxic and nongenotoxic events in rat liver "stem-like" cells. *Toxicology* **2007**, *232*, 147–159, doi:10.1016/j.tox.2006.12.024.

51. Kabátková, M.; Svobodová, J.; Pěnčíková, K.; Mohatad, D.S.; Šmerdová, L.; Kozubík, A.; Machala, M.; Vondráček, J.; Kabatkova, M.; Svobodova, J.; et al. Interactive effects of inflammatory cytokine and abundant low-molecular-weight PAHs on inhibition of gap junctional intercellular communication, disruption of cell proliferation control, and the AhR-dependent transcription. *Toxicol. Lett.* **2015**, *232*, 113–121, doi:10.1016/j.toxlet.2014.09.023.

52. Novakova, K.; Blaha, L.; Babica, P. Tumor promoting effects of cyanobacterial extracts are potentiated by anthropogenic contaminants - Evidence from in vitro study. *Chemosphere* **2012**, *89*, 30–37, doi:10.1016/j.chemosphere.2012.04.008.

53. Luster-Teasley, S.L.; Ganey, P.E.; DiOrio, M.; Ward, J.S. 3rd; Maleczka, R.E.J.; Trosko, J.E.; Masten, S.J. Effect of byproducts from the ozonation of pyrene: biphenyl-2,2′,6,6′-tetracarbaldehyde and biphenyl-2,2′,6,6′-tetracarboxylic acid on gap junction intercellular communication and neutrophil function. *Environ. Toxicol. Chem.* **2005**, *24*, 733–740, doi:10.1897/04-679.1.

54. Herner, H.A.; Trosko, J.E.; Masten, S.J. The epigenetic toxicity of pyrene and related ozonation byproducts containing an aldehyde functional group. *Environ. Sci. Technol.* **2001**, *35*, 3576–3583, doi:10.1021/es0106117.

55. Machala, M.; Svihálková-Sindlerová, L.; Pencíková, K.; Krcmár, P.; Topinka, J.; Milcová, A.; Nováková, Z.; Kozubík, A.; Vondrácek, J. Effects of methylated chrysenes on AhR-dependent and -independent toxic events in rat liver epithelial cells. *Toxicology* **2008**, *247*, 93–101, doi:10.1016/j.tox.2008.02.008.

56. Vondrácek, J.; Chramostová, K.; Plísková, M.; Bláha, L.; Brack, W.; Kozubík, A.; Machala, M.; Vondracek, J.; Chramostova, K.; Pliskova, M.; et al. Induction of aryl hydrocarbon receptor-mediated and estrogen receptor-mediated activities, and modulation of cell proliferation by dinaphthofurans. *Environ. Toxicol. Chem.* **2004**, *23*, 2214–2220, doi:10.1897/03-620.

57. Vondráček, J.; Švihálková-Šindlerová, L.; Pěnčíková, K.; Krčmář, P.; Andrysík, Z.; Chramostová, K.; Marvanová, S.; Valovičová, Z.; Kozubík, A.; Gábelová, A.; et al. 7H-Dibenzo[c,g]carbazole and 5,9-dimethyldibenzo[c,g]carbazole exert multiple toxic events contributing to tumor promotion in rat liver epithelial "stem-like" cells. *Mutat. Res. - Fundam. Mol. Mech. Mutagen.* **2006**, *596*, 43–56, doi:10.1016/j.mrfmmm.2005.11.005.

58. Evans, M.G.; Trosko, J.E. Concentration/response effect of 2,2′, 4,4′, 5,5′-hexabromobiphenyl on cell-cell communication in vitro: assessment by fluorescence redistribution after photobleaching ("FRAP"). *Cell Biol. Toxicol.* **1988**, *4*, 163–171, doi:10.1007/BF00119243.

59. Machala, M.; Bláha, L.; Vondráček, J.; Trosko, J.E.; Scott, J.; Upham, B.L. Inhibition of gap junctional intercellular communication by noncoplanar polychlorinated biphenyls: Inhibitory potencies and screening for potential mode(s) of action. *Toxicol. Sci.* **2003**, *76*, 102–111, doi:10.1093/toxsci/kfg209.

60. Hamers, T.; Kamstra, J.H.; Cenijn, P.H.; Pencikova, K.; Palkova, L.; Simeckova, P.; Vondracek, J.; Andersson, P.L.; Stenberg, M.; Machala, M. In Vitro Toxicity Profiling of Ultrapure Non-Dioxin-like Polychlorinated Biphenyl Congeners and Their Relative Toxic Contribution to PCB Mixtures in Humans. *Toxicol. Sci.* **2011**, *121*, 88–100, doi:10.1093/toxsci/kfr043.

61. Simecková, P.; Vondrácek, J.; Andrysík, Z.; Zatloukalová, J.; Krcmár, P.; Kozubík, A.; Machala, M. The 2,2′,4,4′,5,5′-

hexachlorobiphenyl-enhanced degradation of connexin 43 involves both proteasomal and lysosomal activities. *Toxicol. Sci.* **2009**, *107*, 9–18, doi:10.1093/toxsci/kfn202.

62. Machala, M.; Bláha, L.; Lehmler, H.-J.; Plísková, M.; Májková, Z.; Kapplová, P.; Sovadinová, I.; Vondrácek, J.; Malmberg, T.; Robertson, L.W. Toxicity of hydroxylated and quinoid PCB metabolites: inhibition of gap junctional intercellular communication and activation of aryl hydrocarbon and estrogen receptors in hepatic and mammary cells. *Chem. Res. Toxicol.* **2004**, *17*, 340–347, doi:10.1021/tx030034v.

63. Satoh, A.Y.; Trosko, J.E.; Masten, S.J. Epigenetic toxicity of hydroxylated biphenyls and hydroxylated polychlorinated biphenyls on normal rat liver epithelial cells. *Environ. Sci. Technol.* **2003**, *37*, 2727–2733, doi:10.1021/es021041t.

64. Andrysik, Z.; Prochazkova, J.; Kabatkova, M.; Umannova, L.; Simeckova, P.; Kohoutek, J.J.J.J.J.J.; Kozubik, A.; Machala, M.; Vondracek, J.; Andrysík, Z.; et al. Aryl hydrocarbon receptor-mediated disruption of contact inhibition is associated with connexin43 downregulation and inhibition of gap junctional intercellular communication. *Arch. Toxicol.* **2013**, *87*, 491–503, doi:10.1007/s00204-012-0963-7.

65. Upham, B.L.; Trosko, J.E. A paradigm shift in the understanding of oxidative stress and its implications to exposure of low-level ionizing radiation. *Acta Med. Nagasaki.* **2005**, *50*, 63–68.

66. Oh, S.-Y.Y.; Schmidt, S.A.; Murray, A.W. Epidermal growth factor inhibits Gap junctional communication and stimulates serine-phosphorylation of connexin43 in WB cells by a protein kinase c-independent mechanism. *Cell Commun. Adhes.* **1993**, *1*, 143–149, doi:10.3109/15419069309095690.

67. Upham, B.L.; Kang, K.S.; Cho, H.Y.; Trosko, J.E. Hydrogen peroxide inhibits gap junctional intercellular communication in glutathione sufficient but not glutathione deficient cells. *Carcinogenesis* **1997**, *18*, 37–42, doi:10.1093/carcin/18.1.37.

68. Hasler, C.M.; Frick, M.A.; Bennink, M.R.; Trosko, J.E. TPA-induced inhibition of gap junctional intercellular communication is not mediated through free radicals. *Toxicol. Appl. Pharmacol.* **1990**, *103*, 389–398, doi:10.1016/0041-008x(90)90312-i.

69. Kim, Y.-J.; Seo, S.G.; Choi, K.; Kim, J.E.; Kang, H.; Chung, M.-Y.; Lee, K.W.; Lee, H.J. Recovery Effect of Onion Peel Extract against H2O2-Induced Inhibition of Gap-Junctional Intercellular Communication is Mediated through Quercetin. *J. Food Sci.* **2014**, *79*, H1011–H1017, doi:10.1111/1750-3841.12440.

70. Kim, J.-S.; Ha, T.-Y.; Ahn, J.; Kim, H.-K.; Kim, S. Pterostilbene from Vitis coignetiae protect H2O2-induced inhibition of gap junctional intercellular communication in rat liver cell line. *Food Chem. Toxicol. an Int. J. Publ. Br. Ind. Biol. Res. Assoc.* **2009**, *47*, 404–409, doi:10.1016/j.fct.2008.11.038.

71. Lee, S.J.; Lee, K.W.; Lee, H.J. Abies nephrolepis leaf phenolics prevent the inhibition of gap junction intercellular communication by hydrogen peroxide in rat liver epithelial cells. *Biofactors* **2004**, *21*, 357–360, doi:10.1002/biof.552210168.

72. Hwang, J.-W.; Park, J.-S.; Jo, E.-H.; Kim, S.-J.; Yoon, B.-S.; Kim, S.-H.; Lee, Y.-S.; Kang, K.-S. Chinese cabbage extracts and sulforaphane can protect H2O2-induced inhibition of gap junctional intercellular communication through the inactivation of ERK1/2 and p38 MAP kinases. *J. Agric. Food Chem.* **2005**, *53*, 8205–8210, doi:10.1021/jf051747h.

73. Hwang, J.-W.; Jung, J.-W.; Lee, Y.-S.; Kang, K.-S. Indole-3-Carbinol Prevents H2O2-Induced Inhibition of Gap Junctional Intercellular Communication by Inactivation of PKB/Akt. *J. Vet. Med. Sci.* **2008**, *70*, 1057–1063, doi:10.1292/jvms.70.1057.

74. Kim, J.H.; Choi, S.H.; Kim, J.; Lee, B.K.; Lee, K.W.; Lee, H.J. Differential regulation of the hydrogen-peroxide-induced inhibition of gap-junction intercellular communication by resveratrol and butylated hydroxyanisole. *Mutat. Res.* **2009**, *671*, 40–44, doi:10.1016/j.mrfmmm.2009.08.011.

75. Lee, D.E.; Shin, B.J.; Hur, H.J.; Kim, J.H.; Kim, J.; Kang, N.J.; Kim, D.O.; Lee, C.Y.; Lee, K.W.; Lee, H.J. Quercetin, the active phenolic component in kiwifruit, prevents hydrogen peroxide-induced inhibition of gap-junction intercellular communication. *Br. J. Nutr.* **2010**, *104*, 164–170, doi:10.1017/S0007114510000346.

76. Kim, J.H.; Lee, B.K.; Lee, K.W.; Lee, H.J. Resveratrol counteracts gallic acid-induced down-regulation of gap-junction intercellular communication. *J. Nutr. Biochem.* **2009**, *20*, 149–154, doi:10.1016/j.jnutbio.2008.01.008.

77. Jung, J.-W.; Cho, S.-D.; Ahn, N.-S.; Yang, S.-R.; Park, J.-S.; Jo, E.-H.; Hwang, J.-W.; Aruoma, O.I.; Lee, Y.-S.; Kang, K.-S. Effects of the histone deacetylases inhibitors sodium butyrate and trichostatin A on the inhibition of gap junctional intercellular communication by H2O2- and 12-O-tetradecanoylphorbol-13-acetate in rat liver epithelial cells. *Cancer Lett.* **2006**, *241*, 301–308, doi:10.1016/j.canlet.2005.10.029.

78. Lee, K.W.; Hur, H.J.; Lee, H.J.; Lee, C.Y.; Ki, W.L.; Haeng, J.H.; Hyong, J.L.; Chang, Y.L.; Lee, K.W.; Hur, H.J.; et al. Antiproliferative effects of dietary phenolic substances and hydrogen peroxide. *J. Agric. Food Chem.* **2005**, *53*, 1990–1995, doi:10.1021/jf0486040.

79. Ki, W.L.; Hwang, E.-S.E.-S.; Nam, J.K.; Kyoung, H.K.; Hyong, J.L.; Lee, K.W.; Hwang, E.-S.E.-S.; Kang, N.J.; Kim, K.H.; Lee, H.J.; et al. Extraction and chromatographic separation of anticarcinogenic fractions from cacao bean husk. *BioFactors* **2005**, *23*, 141–150, doi:10.1002/biof.5520230303.

80. Kim, J.-S.; Lee, W.-M.; Rhee, H.C.; Kim, S. Red paprika (Capsicum annuum L.) and its main carotenoids, capsanthin and β-carotene, prevent hydrogen peroxide-induced inhibition of gap-junction intercellular communication. *Chem. Biol. Interact.* **2016**, *254*, 146–155, doi:10.1016/j.cbi.2016.05.004.

81. Cho, J.-H.; Cho, S.-D.; Hu, H.; Kim, S.-H.; Lee, S.K.; Lee, Y.-S.; Kang, K.-S. The roles of ERK1/2 and p38 MAP kinases in the preventive mechanisms of mushroom Phellinus linteus against the inhibition of gap junctional intercellular communication by hydrogen peroxide. *Carcinogenesis* **2002**, *23*, 1163–1169, doi:10.1093/carcin/23.7.1163.

82. Hu, W.; Jones, P.D.; Upham, B.L.; Trosko, J.E.; Lau, C.; Giesy, J.P. Inhibition of gap junctional intercellular communication by perfluorinated compounds in rat liver and dolphin kidney epithelial cell lines in vitro and Sprague-Dawley rats in vivo. *Toxicol. Sci.* **2002**, *68*, 429–436, doi:10.1093/toxsci/68.2.429.

83. Upham, B.L.; Park, J.-S.; Babica, P.; Sovadinova, I.; Rummel, A.M.; Trosko, J.E.; Hirose, A.; Hasegawa, R.; Kanno, J.; Sai, K. Structure-activity-dependent regulation of cell communication by perfluorinated fatty acids using in vivo and in vitro model systems. *Environ. Health Perspect.* **2009**, *117*, 545–551, doi:10.1289/ehp.11728.

84. Madhukar, B. V; de Feijter-Rupp, H.L.; Trosko, J.E. Pulse treatment with the tumor promoter TPA delays the onset of desensitization response and prolongs the inhibitory effect on gap junctional intercellular communication of a rat liver epithelial cell line WB F-344. *Cancer Lett.* **1996**, *106*, 117–123, doi:10.1016/0304-3835(96)04315-7.

85. Sun, H.; Liu, G.-T. Chemopreventive effect of bicyclol on malignant transformation of WB-F344 rat liver epithelial cells and its effect on related signal transduction in vitro. *Cancer Lett.* **2006**, *236*, 239–249, doi:10.1016/j.canlet.2005.05.019.

86. Kang, K.S.; Yun, J.W.; Yoon, B.; Lim, Y.K.; Lee, Y.S. Preventive effect of germanium dioxide on the inhibition of gap junctional intercellular communication by TPA. *Cancer Lett.* **2001**, *166*, 147–153, doi:10.1016/s0304-3835(01)00446-3.

87. Sun, H.; Liu, G. Chemopreventive effect of dimethyl dicarboxylate biphenyl on malignant transformation of WB-F344 rat liver epithelial cells. *Acta Pharmacol. Sin.* **2005**, *26*, 1339–1344, doi:10.1111/j.1745-7254.2005.00208.x.

88. Oh, S.Y.; Madhukar, B. V; Trosko, J.E. Inhibition of gap junctional blockage by palmitoyl carnitine and TMB-8 in a rat liver epithelial cell line. *Carcinogenesis* **1988**, *9*, 135–139, doi:10.1093/carcin/9.1.135.

89. Park, J.-R.; Park, J.-S.; Jo, E.-H.; Hwang, J.-W.; Kim, S.-J.; Ra, J.-C.; Aruoma, O.I.; Lee, Y.-S.; Kang, K.-S. Reversal of the TPA-induced inhibition of gap junctional intercellular communication by Chaga mushroom (Inonotus obliquus) extracts: effects on MAP kinases. *Biofactors* **2006**, *27*, 147–155, doi:10.1002/biof.5520270113.

90. Prochazka, L.; Turanek, J.; Tesarik, R.; Knotigova, P.; Polaskova, P.; Andrysik, Z.; Kozubik, A.; Zak, F.; Sova, P.; Neuzil, J.; et al. Apoptosis and inhibition of gap-junctional intercellular communication induced by LA-12, a novel hydrophobic platinum(IV) complex. *Arch. Biochem. Biophys.* **2007**, *462*, 54–61, doi:10.1016/j.abb.2007.03.021.

91. Čtveráčková, L.; Jančula, D.; Raška, J.; Babica, P.; Sovadinová, I. Structure-Dependent Effects of Phthalates on Intercellular and Intracellular Communication in Liver Oval Cells. *Int. J. Mol. Sci.* **2020**, *21*, doi:10.3390/ijms21176069.

92. Ye, Y.-X.; Bombick, D.; Hirst, K.; Zhang, G.; Chang, C.-C.; Trosko, J.E.; Akera, T. The modulation of gap junctional

communication by Gossypol in various mammalian cell lines in vitro. *Toxicol. Sci.* **1990**, *14*, 817–832, doi:10.1093/toxsci/14.4.817.

93. Suzuki, J.; Na, H.K.; Upham, B.L.; Chang, C.C.; Trosko, J.E. lambda-Carrageenan-induced inhibition of gap-junctional intercellular communication in rat liver epithelial cells. *Nutr. CANCER-AN Int. J.* **2000**, *36*, 122–128, doi:10.1207/S15327914NC3601_16.

94. Upham, B.L.; Koski, T.R.; Rummel, A.M.; Wilson, M.R.; Horvath, A.; Trosko, J.E. Differential roles of 2, 6, and 8 carbon ceramides on the modulation of gap junctional communication and apoptosis during carcinogenesis. *Cancer Lett.* **2003**, *191*, 27–34, doi:10.1016/s0304-3835(02)00620-1.
